# Physical and cognitive contributions to fatigue perception: The interplay between local muscle fatigue and sensory prediction error

**DOI:** 10.1016/j.isci.2026.115552

**Published:** 2026-04-01

**Authors:** Zihang Xu, Baichun Wei, Chifu Yang, Haiqi Zhu, Chunyu Zhang, Zhiyuan Chen, Shuqing Chen, Chunzhi Yi

**Affiliations:** 1 School of Mechatronics Engineering, Harbin Institute of Technology, Harbin 150001, China; 2 School of Medicine and Health, Harbin Institute of Technology, Harbin 150001, China; 3 School of Sport Medicine and Rehabilitation, Beijing Sport University, Beijing 100084, China; 4 School of Computer Science, Faculty of Science and Engineering, University of Nottingham Malaysia, Jalan Broga, Semenyih 43500, Selangor, Malaysia; 5 Zhengzhou Research Institute, Harbin Institute of Technology, Harbin, China; 6 Suzhou Research Institute, Harbin Institute of Technology, Harbin, China; 7 Institute for Neural Information Processing, University Medical Center Hamburg- Eppendorf, Hamburg, Germany

**Keywords:** Neuroscience, Behavioral neuroscience, Sensory neuroscience, Cognitive neuroscience

## Abstract

Fatigue perception during exercise arises from complex body-brain interactions, but integration of local muscle fatigue with sensory prediction errors remains unclear. Traditional cognitive frameworks overlook dynamic physiological contributions. This study examined how local muscle fatigue and prediction errors jointly shape fatigue perception across spatial-temporal domains. Two experiments used naturalistic running with physiological monitoring, inducing temporal and spatial prediction errors by manipulating performance feedback. Computational models quantified contributions of muscle fatigue, prediction errors, and their interactions. Results showed fatigue perception is driven by both muscle fatigue and prediction errors, with domain-specific interactions: temporal errors linearly amplified muscle fatigue’s impact, while spatial errors modulated it exponentially. These findings challenge purely cognitive models, demonstrating that fatigue perception emerges from domain-dependent integration of physiological signals and sensory discrepancies. The study provides a unified computational framework for body-brain interactions in fatigue, offering insights for personalized training and rehabilitation targeting both physical and cognitive fatigue pathways.

## Highlights

• The study provides a computational framework for brain-body interaction in fatigue
• Fatigue perception integrates sensory prediction errors and local muscle fatigue
• Sensory prediction errors in different domains affect muscle fatigue differently
• Temporal error effect is directional, and spatial error effect is non-directional

## Graphical abstract

**Figure:**
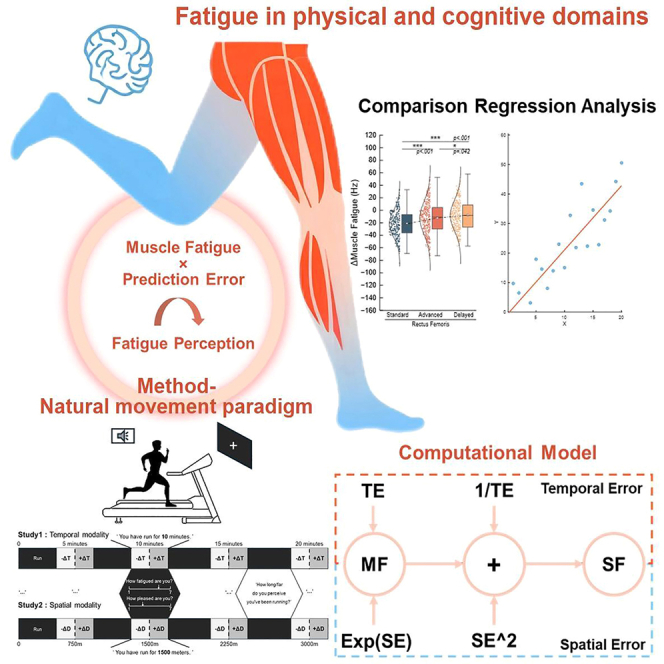

## Introduction

Prolonged exercise can induce fatigue, a comprehensive state involving subjective feeling of exhaustion and physical decline of local muscle strength or endurance,^1^^,^^2^ which would degrade exercise performance.^3^ Despite the subjective fatigue perception and physical muscle fatigue are accompanied, previous studies indicated that the two kinds of fatigue present a complex coupling with each other, which varied under specific tasks. For example, during low-load contractions task, right trapezius muscle presents a non-simultaneous changes with the subjective fatigue perception.^4^ Other studies indicated that some factors, like sensory feedback,^5^ pathological states^6^ and exercises,^7^ have differential impacts on fatigue perception and muscle fatigue, which suggested that potentially different mechanisms of the two kinds of fatigue. Understanding how fatigue perception arises and how it interacts with muscle fatigue would benefit determining principles of training dose and improving motor performance, given that fatigue perception limits endurance and performance of exercise.^8^ Previous studies especially focused on the cognitive domain and showed that fatigue perception associated with the error between motor prediction and output,^5^ reward,^9^ potential efforts being paid.^10^ Given the theoretical perspective on the body-brain interaction of fatigue perception,^4^ crucial questions, however, still remain on integrating bodily states and cognitive aspects to understand the temporal dynamics of fatigue perception. Firstly, does fatigue perception associate with muscle states or fatigue? And secondly, what is the computational mechanism of the association among sensory feedback, muscle fatigue and fatigue perception?

Previous studies and reviews have provided some insights into these questions. It was suggested that the subjective fatigue perception induced by prolonged exercise had a strong link with the sensory prediction error, i.e., the discrepancy between expectation and actual performance of motions, which increased the feelings of efforts.^5^^,^^11^^,^^12^ This has been thought to associate with the discrepancy between the efferent copy generated before action execution and the sensory feedback during actual action.^13^^,^^14^^,^^15^ However, theoretical accounts posit that other than the sensor prediction error in the cognitive domain, local muscle fatigue in the physical domain may also contribute to the dynamics of fatigue perception.^16^^,^^17^^,^^18^^,^^19^ Few studies have simultaneously assessed the sensory prediction error, fatigue perception and local muscle fatigue during prolonged exercises, despite being a cornerstone of understanding the interaction between cognitive and physical domains during fatigue.^20^^,^^21^ Thus, whether fatigue perception during prolonged exercise is associated with muscle fatigue, how sensor prediction error and muscle fatigue interact with each other and the computational processes underpinning the progress, is unknown.

Although debatable, previous studies have provided cues on how local muscle fatigue and sensory prediction error may associate with fatigue perception. Performed time manipulation and measured the moment-by-moment fluctuations of electromyography (EMG) and fatigue perception. Their results suggested that the artificially introduced sensory prediction error in the temporal modality induced significant changes of fatigue perception,^5^ and the EMG of the flexor showed different median frequency across different delay time. Matta et al.^22^ indicated the sensory prediction error induced by time manipulation had a coupling with muscle fatigue, suggesting a potential interaction between sensory prediction error in the cognitive domain and muscle fatigue in the physical domain. However, the study of A. Steens et al.,^6^ which investigated the association among fatigue perception, maximum voluntary contraction force and force decline, showed that the fatigue perception only significantly related with muscle fatigue in multiple sclerosis patients, not in healthy controls. Despite the emphasis on including muscular fatigue, the association between fatigue perception and motor sensory changes of local muscles are largely unexplored.

**Figure 1:**
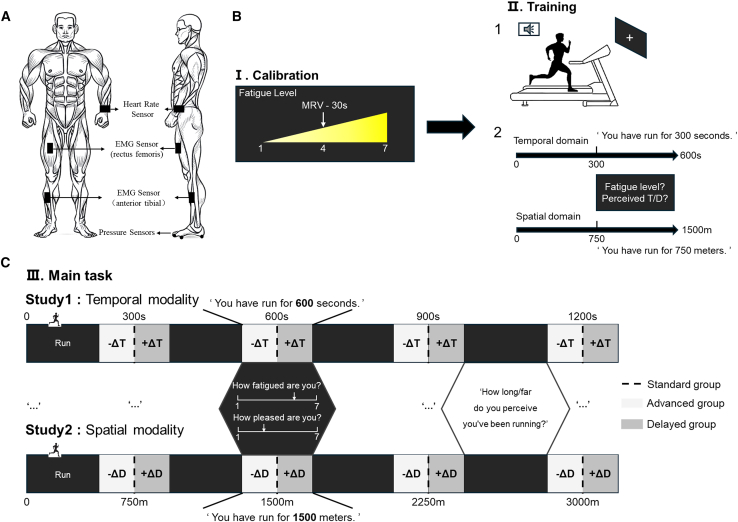
Experimental protocol (A) Diagram of the installation of wireless EMG sensors (the right rectus femoris and anterior tibialis), heart rate monitoring smart bracelet (the left wrist) and pressure sensors (the heel and first metatarsal bone of the left and right feet) for the experiment. (B) Calibration and training process for study 1 (temporal modality) and study 2 (spatial modality). In the calibration part, participants determined maximum running velocity (MRV) by increasing treadmill speed until instability for 30 s, setting the fatigue perception at that time as level 4. Main task speed was 70% MRV. Training phase involved treadmill familiarization and main task procedure explanation. A 10-min or 1,500-m training in each modality ensured they knew the procedure well. After each block, participants reported a fatigue/pleasure level; the final block included a perception question. (T represents time and D represents distance). (C) Trial structure in the main task. Participants in study 1/2 were verbally told each block was 300 s/750 m. Unlike training, actual time/distance varied across groups: standard groups received accurate info, advanced groups got pre-emptive verbal feedback, and delayed groups received it after achieving the target. After each block, participants also needed to report a fatigue/pleasure level; the final block included a question about perceived running time or distance.

In our study, we answer these questions by investigating the computational processes of how sensory prediction error in temporal or spatial modality and muscle fatigue associate with subjective fatigue perception. We designed a time/distance-based exercise paradigm (Figure 1) where participants performed treadmill running while receiving manipulated performance feedback with EMG and heart rate monitoring. The main experimental phase consisted of four running blocks per study: 5-min duration blocks for study 1(temporal modality) and 750-meter distance blocks for study 2 (spatial modality). Crucially, while all participants received standardized verbal updates about their “completed” time/distance (5 min or 750 m per block), their actual running performance was systematically manipulated across three experimental groups without their awareness. In the standard group, computer-generated verbal updates matched real performance metrics (every 5 min/750 m). In the advanced group, participants received updates before actually reaching target time or distance (sort randomly from [4.5 min, 4.0 min, 3.5 min, 3.0 min]/[675 m, 600 m, 525 m, 450 m]), while in the delayed group participants received them later than actually achieving time or distance (sort randomly from [5.5 min, 6.0 min, 6.5 min, 7.0 min]/[825 m, 900 m, 975 m, 1,050 m]). When each block finished, we collected participants’ fatigue and pleasure levels. Each participant was involved in three experimental groups. The order of the experiments was random, and there was a one-week interval between each experiment to ensure that the participants’ fatigue had completely recovered.

Compared with the experimental paradigms of previous studies that used grip strength, isometric or isokinetic contraction, we used running with self-determined speed as the test bed, which is closer to the natural scenario of daily exercise. We performed group-based experiments with different sensory prediction errors in both temporal and spatial modalities and assessed the temporal progression of muscle fatigue and fatigue perception. We investigated why groups with different sensory prediction errors presented similar levels of fatigue perception by associating with muscle fatigue. We further tested the computational processes of sensory prediction error, muscle fatigue and fatigue perception separately in temporal and spatial modalities by performing model comparison. We provided a computational framework that could explain the temporal progression of fatigue perception in different modalities, highlighting muscle fatigue in the physical domain can be coupled with sensory prediction error and directly linked to fatigue perception in the cognitive domain.

## Results

### Exercise significantly changed the psychological and physiological factors of the subjects

**Figure 2:**
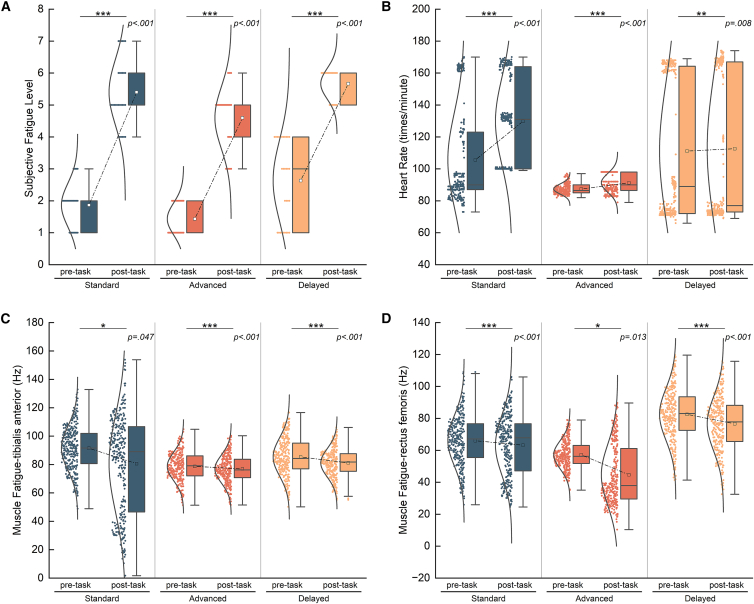
Changes in physiological and psychological factors in temporal modality (A) Changes in the subjective fatigue perception. We used paired sample *t* test in this section. The subjective fatigue perception level significantly increased after the task. (Standard: DF = 29, *p* < 0.001, Cohen’s d = 2.65, 95%CI = [3.07, 4.00], advanced: DF = 29, *p* < 0.001, Cohen’s d = 3.47, 95%CI = [2.87, 3.50], delayed: DF = 29, *p* < 0.001, Cohen’s d = 2.24, 95%CI = [2.60, 3.53]). (B) Changes in the heart rate. The heart rate significantly increased after the task. (Standard: DF = 299, *p* < 0.001, Cohen’s d = 1.19, 95%CI = [21.88, 26.50], advanced: DF = 288, *p* < 0.001, Cohen’s d = 0.53, 95%CI = [2.99, 4.68], delayed: DF = 299, *p* = 0.008, Cohen’s d = 0.15, 95%CI = [0.41, 2.63]). (C) Changes in the median frequency of tibialis anterior. (Standard: DF = 292, *p* = 0.047, Cohen’s d = −0.12, 95%CI = [−5.39, −0.05], advanced: DF = 292, *p* < 0.001, Cohen’s d = −0.56, 95%CI = [−15.20, −10.00], delayed: DF = 292, *p* < 0.001, Cohen’s d = −0.35, 95%CI = [−7.88, −3.94]). (D) Changes in the median frequency of rectus femoris. The median frequency of two muscles significantly decreased. (Standard: DF = 299, *p* < 0.001, Cohen’s d = −0.38, 95%CI = [−14.11, −7.68], advanced: DF = 292, *p* = 0.013, Cohen’s d = −0.15, 95%CI = [−3.28, −0.42], delayed: DF = 279, *p* < 0.001, Cohen’s d = −0.27, 95%CI = [−5.98, −2.37]).

**Figure 3:**
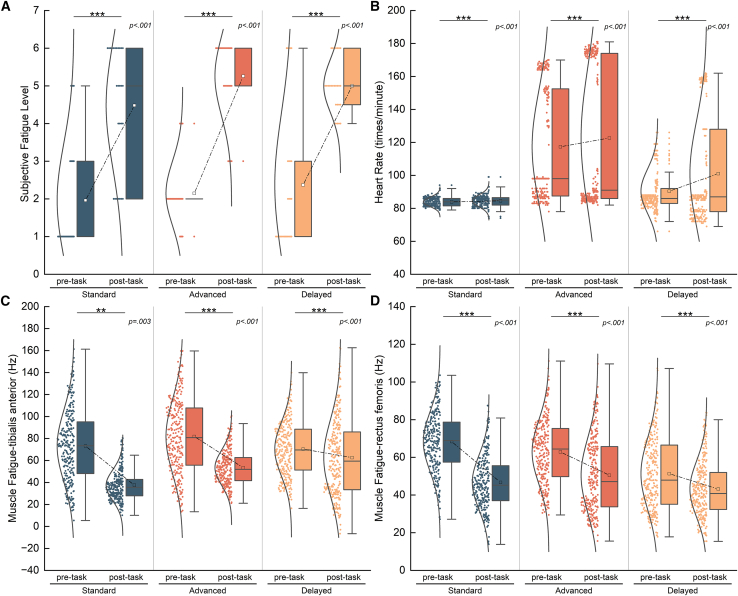
Changes in physiological and psychological factors in spatial modality (A) Changes in the subjective fatigue perception. We used paired sample *t* test in this section. The subjective fatigue perception level significantly increased after the task. (Standard: DF = 26, *p* < 0.001, Cohen’s d = 1.11, 95%CI = [1.67, 3.37], advanced: DF = 26, *p* < 0.001, Cohen’s d = 1.67, 95%CI = [2.41, 3.74], delayed: DF = 26, *p* < 0.001, Cohen’s d = 1.04, 95%CI = [1.69, 3.52]). (B) Changes in the heart rate. The heart rate significantly increased after the task. (Standard: DF = 231, *p* = 0.003, Cohen’s d = 0.19, 95%CI = [0.22, 1.11], advanced: DF = 269, *p* < 0.001, Cohen’s d = 0.36, 95%CI = [3.58, 7.16], delayed: DF = 269, *p* < 0.001, Cohen’s d = 0.31, 95%CI = [6.57, 14.76]). (C) Changes in the median frequency of tibialis anterior. (Standard: DF = 269, *p* < 0.001, Cohen’s d = −1.02, 95%CI = [−24.155, −19.155], advanced: DF = 269, *p* < 0.001, Cohen’s d = −0.47, 95%CI = [−15.46, −9.19], delayed: DF = 269, *p* < 0.001, Cohen’s d = −0.34, 95%CI = [−10.95, −5.23]). (D) Changes in the median frequency of rectus femoris. The median frequency of two muscles significantly decreased. (Standard: DF = 269, *p* < 0.001, Cohen’s d = −1.03, 95%CI = [−39.91, −31.63], advanced: DF = 269, *p* < 0.001, Cohen’s d = −0.76, 95%CI = [−32.84, −23.92], delayed: DF = 269, *p* < 0.001, Cohen’s d = −0.27, 95%CI = [−11.21, −4.29]).

We firstly tested whether the prolonged running induces changes of local muscle fatigue, fatigue perception and heart rate. We compared the median frequency of rectus femoris and tibialis anterior, heart rate and scales of fatigue perception before and after the whole session for each group (Figures 2 and 3). We found that the subjective fatigue perception levels in groups of two modalities all showed an increase after exercise and the average heart rate increased and the median frequency of EMG decreased, indicating that the setting of the exercise tasks effectively induced changes in the subjective fatigue state and the peripheral fatigue state.

**Figure 4:**
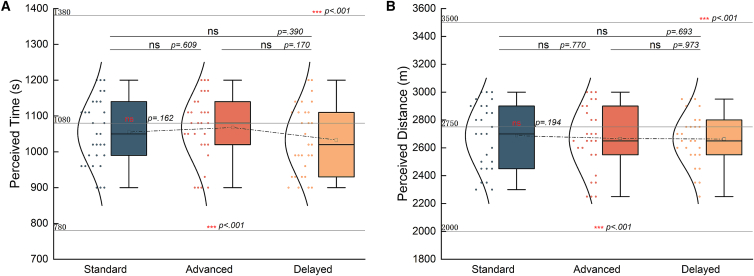
Results of comparing perceived processes among groups in spatiotemporal modality (A) Comparison of the perceived exercise duration and the actual duration in temporal modality. The horizontal reference lines represent the actual exercise duration of three groups during the inquiry, which are 1,080 s, 780 s, and 1,380 s, respectively. We compared the differences in perceived exercise duration and actual duration (red symbol, standard: DF = 29, *p* = 0.162, effect = −0.34, 95%CI = [−55.00, 6.03], advanced: DF = 29, *p* < 0.001, effect = −0.87, 95%CI = [257.00, 319.00], delayed: DF = 29, *p* < 0.001, effect = −0.87, 95%CI = [−379.00, −313.98]), and compared the differences between groups. (Black symbol, standard: DF = 58, *p* = 0.609, Cohen’s d = −0.14, 95%CI = [−59.00, 33.00], advanced: DF = 58, *p* = 0.390, Cohen’s d = 0.23, 95%CI = [−26.00, 69.00], delayed: DF = 58, *p* = 0.170, Cohen’s d = 0.37, 95%CI = [−13.00, 81.00]). (B) Comparison of perceived exercise duration among groups in spatial modality. (single sample test: standard: DF = 26, *p* = 0.194, effect = −0.30, 95%CI = [−153.70, 20.42], advanced: DF = 26, *p* < 0.001, effect = −0.87, 95%CI = [581.44, 753.70], delayed: DF = 26, *p* < 0.001, effect = −0.87, 95%CI = [−903.75, −768.52]; permutation test: standard: DF = 52, *p* = 0.770, Cohen’s d = −0.09, 95%CI = [−103.75, 144.44], advanced: DF = 52, *p* = 0.693, Cohen’s d = 0.11, 95%CI = [−87.04, 135.19], delayed: DF = 52, *p* = 0.973, Cohen’s d = 0.02, 95%CI = [−109.26, 116.67]).

We then examined whether the sensory prediction errors were successfully induced. To do so, we compared between the perceived exercise duration/distance and the actual exercise duration/distance of subjects in the three groups of two modalities (Figure 4). In the standard group, there was no significant difference between the perceived and actual states, indicating that in the experiment, the subjects could reasonably perceive the exercise process under the guidance of the prompt sound. In the advanced group, the perceived duration/distance was more delayed than the actual one. Conversely, the delayed group was just the opposite. This phenomenon demonstrates that we successfully induced different sensory prediction errors through the manipulation of spatiotemporal errors in different directions. We then evaluated whether the subjects identified each group by the perceived duration or distance. We compared the subjects’ perceived exercise process between groups. The results showed that the subjects did not notice the differences across the experimental conditions among different groups (Figure 4).

**Figure 5:**
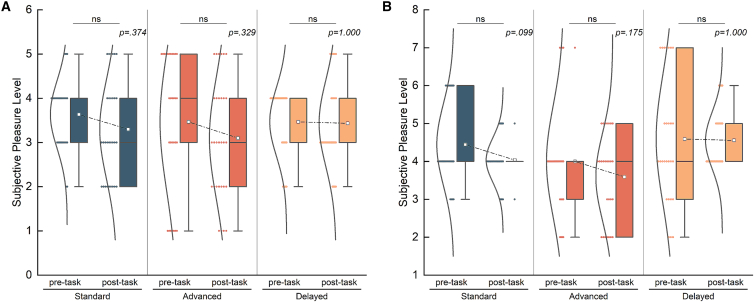
Results of comparing the subjective pleasure level between pre- and post-task in spatiotemporal modality (A) Comparison of the changes of pleasure level in temporal modality. (Standard: DF = 29, *p* = 0.374, Cohen’s d = −0.18, 95%CI = [−0.97, 0.33], advanced: DF = 29, *p* = 0.329, Cohen’s d = −0.20, 95%CI = [−1.03, 0.27], delayed: DF = 29, *p* = 1.000, Cohen’s d = −0.03, 95%CI = [−0.50, 0.43]). (B) Comparison of the changes of pleasure level in spatial modality. (Standard: DF = 26, *p* = 0.099, Cohen’s d = −0.36, 95%CI = [−0.82, 0.00], advanced: DF = 26, *p* = 0.175, Cohen’s d = −0.29, 95%CI = [−0.93, 0.07], delayed: DF = 26, *p* = 1.000, Cohen’s d = −0.02, 95%CI = [−0.85, 0.74]).

To eliminate the effect of pleasure derived from running on the experimental results, we compared the changes in the subjects’ pleasure levels before and after exercise, there were no significant differences in the pleasure perception levels (Figure 5), indicating that the experimental paradigm did not cause changes in the pleasure perception levels.

### Why the fatigue perception presented no significant difference across groups?

**Figure 6:**
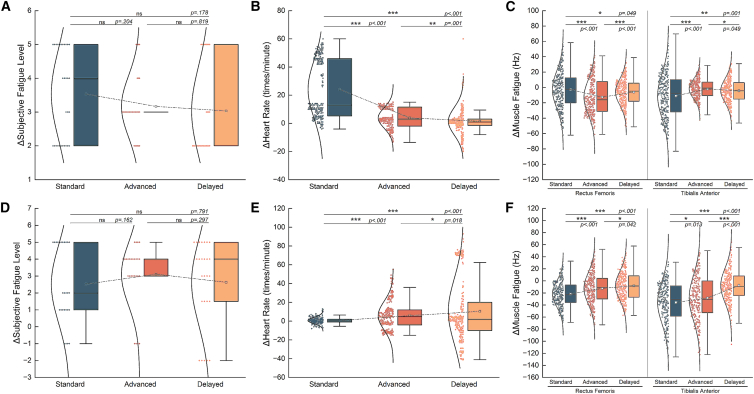
Comparison of the ranges of changes in physiological and psychological factors among groups in two studies (A) Comparison of the changes of perceived fatigue level in temporal modality. (Standard vs. advanced: DF = 56, *p* = 0.204, Cohen’s d = 0.36, 95%CI = [−0.14, 1.00], standard vs. delayed: DF = 56, *p* = 0.178, Cohen’s d = 0.39, 95%CI = [−0.17, 1.21], advanced vs. delayed: DF = 56, *p* = 0.819, Cohen’s d = 0.09, 95%CI = [−0.45, 0.66]). (B) Comparison of the changes of heart rate in temporal modality. (Standard vs. advanced: DF = 585, *p* < 0.001, Cohen’s d = 0.36, 95%CI = [18.04, 22.69], standard vs. delayed: DF = 596, *p* < 0.001, Cohen’s d = 0.39, 95%CI = [20.41, 25.18], advanced vs. delayed: DF = 585, *p* = 0.001, Cohen’s d = 0.09, 95%CI = [0.95, 3.74]). (C) Comparison of the changes of muscle fatigue level in temporal modality. (Rectus Femoris: standard vs. advanced: DF = 582, *p* < 0.001, Cohen’s d = 0.43, 95%CI = [6.23, 13.77], standard vs. delayed: DF = 580, *p* = 0.049, Cohen’s d = 0.17, 95%CI = [0.28, 6.81], advanced vs. delayed: DF = 580, *p* < 0.001, Cohen’s d = −0.33, 95%CI = [−9.82, −3.14], Tibialis Anterior: standard vs. advanced: DF = 589, *p* < 0.001, Cohen’s d = −0.41, 95%CI = [−12.31, −5.39], standard vs. delayed: DF = 576, *p* = 0.001, Cohen’s d = −0.28, 95%CI = [−10.32, −2.89], advanced vs. delayed: DF = 569, *p* = 0.049, Cohen’s d = 0.17, 95%CI = [0.01, 4.58]). (D) Comparison of the changes of perceived fatigue level in spatial modality. (Standard vs. advanced: DF = 498, *p* < 0.001, Cohen’s d = −0.43, 95%CI = [−1.96, 0.19], standard vs. delayed: DF = 498, *p* < 0.001, Cohen’s d = −0.40, 95%CI = [−1.40, 1.14], advanced vs. delayed: DF = 536, *p* = 0.018, Cohen’s d = −0.20, 95%CI = [−0.40, 1.89]). (E) Comparison of the changes of heart rate in spatial modality. (Standard vs. advanced: DF = 56, *p* = 0.204, Cohen’s d = 0.36, 95%CI = [−6.58, −2.89], standard vs. delayed: DF = 56, *p* = 0.178, Cohen’s d = 0.39, 95%CI = [−14.16, −6.05], advanced vs. delayed: DF = 56, *p* = 0.819, Cohen’s d = 0.09, 95%CI = [−9.82, −1.07]). (F) Comparison of the changes of muscle fatigue level in temporal modality. (Rectus Femoris: standard vs. advanced: DF = 535, *p* < 0.001, Cohen’s d = −0.39, 95%CI = [−13.04, −5.15], standard vs. delayed: DF = 536, *p* < 0.001, Cohen’s d = −0.60, 95%CI = [−17.44, −9.74], advanced vs. delayed: DF = 535, *p* = 0.042, Cohen’s d = −0.18, 95%CI = [−9.04, −0.42], Tibialis Anterior: standard vs. advanced: DF = 536, *p* = 0.013, Cohen’s d = −0.21, 95%CI = [−14.15, −1.30], standard vs. delayed: DF = 536, *p* < 0.001, Cohen’s d = −0.88, 95%CI = [−33.21, −22.49], advanced vs. delayed: DF = 536, *p* < 0.001, Cohen’s d = −0.61, 95%CI = [−26.22, −14.58]).

We compared the change of fatigue perception levels across the standard, advanced and delayed groups for temporal and spatial modalities, respectively. We found there was no significance between each pair of the three groups (Figures 6A and 6D), which violates previous theory on subjective fatigue^23^ and previous results within temporal modality.^5^ We then asked why there is no significant difference. We found there is significant difference for the change of both heart rate and median frequency of the two muscles across the three groups (Figures 6B, 6C, 6E, and 6F), and the fairly difference sensory prediction errors among the groups induced by experimental setup. Specifically, we asked whether this result was due to the interacted contributions between sensory prediction error and muscle fatigue, or the fact that the sensory prediction errors were too small to induce significant difference, or the influence of confounding factors.

**Figure 7:**
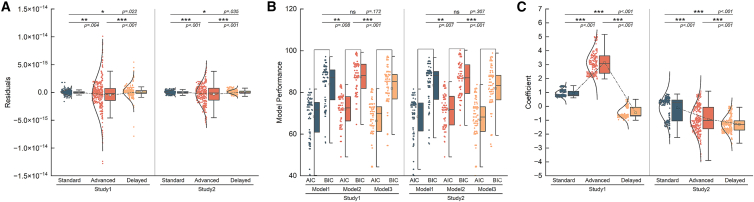
Comparison results of the statistical regression analysis (A) Results of the residuals of the partial regression models among groups in the spatiotemporal modality. (Study1: standard vs. advanced: DF = 716, *p* = 0.004, Cohen’s d = 0.22, 95%CI = [1.57e−16, 8.04e−16], standard vs. delayed: DF = 716, *p* = 0.022, Cohen’s d = −0.17, 95%CI = [−2.59e−16, −1.85e−17], advanced vs. delayed: DF = 716, *p* < 0.001, Cohen’s d = −0.26, 95%CI = [−9.59e−16, −2.64e−16], study2: standard vs. advanced: DF = 602, *p* < 0.001, Cohen’s d = −0.27, 95%CI = [1.48e−16, 5.53e−16], standard vs. delayed: DF = 577, *p* = 0.035, Cohen’s d = −0.18, 95%CI = [−1.00e−16, −1.82e−18], advanced vs. delayed: DF = 581, *p* < 0.001, Cohen’s d = −0.31, 95%CI = [−6.20e−16, −2.02e−16]). (B) Results of the performance of regression models with different combinations of independent variables in the spatiotemporal modality. (Study1: model1 vs. model2: DF = 196, *p* = 0.008, Cohen’s d = −0.40, 95%CI = [−5.87, −0.89], model1 vs. model3: DF = 195, *p* = 0.172, Cohen’s d = 0.19, 95%CI = [−0.79, 5.02], model2 vs. model3: DF = 195, *p* < 0.001, Cohen’s d = 0.57, 95%CI = [2.86, 8.22], Study2: model1 vs. model2: DF = 196, *p* = 0.007, Cohen’s d = −0.37, 95%CI = [−5.77, −1.11], model1 vs. model3: DF = 196, *p* = 0.307, Cohen’s d = 0.14, 95%CI = [−1.33, 4.56], model2 vs. model3: DF = 196, *p* < 0.001, Cohen’s d = 0.51, 95%CI = [2.15, 7.7]). (C) Results of the regression coefficients of muscle fatigue level in spatiotemporal modality. (Study1: standard vs. advanced: DF = 481, *p* < 0.001, Cohen’s d = −4.01, 95%CI = [−2.32, −2.08], standard vs. delayed: DF = 580, *p* < 0.001, Cohen’s d = 4.53, 95%CI = [1.35, 1.45], advanced vs. delayed: DF = 485, *p* < 0.001, Cohen’s d = 6.18, 95%CI = [3.48, 3.73], study2: standard vs. advanced: DF = 558, *p* < 0.001, Cohen’s d = 0.92, 95%CI = [0.67, 0.95], standard vs. delayed: DF = 550, *p* < 0.001, Cohen’s d = 1.70, 95%CI = [1.07, 1.30], advanced vs. delayed: DF = 550, *p* < 0.001, Cohen’s d = 0.47, 95%CI = [0.24, 0.5]).

We tested whether the non-significant difference is because the sensory prediction errors we induced were too small. In this section, we used the first principal component of the median frequencies of the two muscles from PCA as the measurement of muscle fatigue in two studies. We first performed partial regression. Specifically, we regressed the increment of heart rate, median frequency of two muscles and subjective pleasure levels after minus before experiment of each subject against the increment of subjective fatigue perception, respectively for each of the three groups (multi-variable linear regression) of each modality. Then, we compared the residuals across the three groups of different modalities. As shown in Figure 7A, the residuals present difference among the three groups in both temporal and spatial modalities, suggesting that after regressing out the influence of other factors, the influence of the sensory prediction errors is significant. We further confirmed this by performing model comparison respectively in two modalities. Specifically, we performed three regressions: (1) regressing conditions (advanced group = 1, standard group = 2, delayed group = 3), the increment of heart rate, median frequency of two muscles and subjective pleasure levels after minus before experiment of each subject against the increment of subjective fatigue perception (model 1); (2) deleting conditions from model 1 (model 2); and (3) deleting the increment of heart rate from model 1 (model 3). Then, we compared the fitting performance of the three models by Akaike Information Criterion (AIC) and the Bayesian Information Criterion (BIC). As shown in Figure 7B, the fitting performance of model 1 is better than that of model 2, indicating that including conditions can account better for the subjective fatigue perception. The fitting performance of model 1 and 3 is similar, indicating that including the increment of heart rate or not does not harm the fitting results. Overall, the results suggested that the sensory prediction errors of different groups induced different variations of subjective fatigue perception.

We further asked whether the non-significantly different subjective fatigue perception across groups is associated with both sensory prediction errors and muscle fatigue. We regressed conditions, the increment of muscle fatigue, heart rate and subjective pleasure perception levels after minus before experiments, and the interactions among each of these items against the increment of subjective fatigue perception. As the result of study 1, we observed a significant main effect of experimental conditions (*p* = 0.039, estimate = −1.345), muscle fatigue change (*p* = 0.036, estimate = 1.903) and heart rate change (*p* = 0.048, estimate = 0.521) that was moderated by a two-way interaction between experimental conditions and muscle fatigue change (*p* = 0.023, estimate = −3.058). All remaining effects and interactions were not significant. The results demonstrated that the changes in the subjective fatigue perception were associated with the changes of muscle fatigue, heart rate, and experiment conditions, not related to the pleasure perception and its interactions with other variables.

As for study 2, we observed a significant main effect of pleasure × experimental conditions (*p* = 0.019, estimate = 0.740), experimental conditions × heart rate change (*p* = 0.002, estimate = −1.464) and muscle fatigue change × heart rate change (*p* = 0.018, estimate = 2.058). All remaining effects and interactions were not significant. The results indicated that the changes in subjective fatigue perception caused by exercises over a certain distance were related to the interaction between the sensory prediction error and the heart rate as well as the interaction between the change in muscle fatigue degree and the change in heart rate. However, there were no significant changes in the participants’ pleasure perception before and after the experiment, and pleasure alone as the main effect did not have a significant effect on subjective fatigue perception. The significance of the linear regression model might not represent a real strong relationship between the interaction of pleasure perception × the sensory prediction error and subjective fatigue perception. It might be the result of potential confounding factors between the two. Based on this observation, we considered removing pleasure perception in the subsequent computational models and focusing on the other variables that we were concerned about.

We then asked how the spatial/temporal sensory prediction errors would influence the association between muscle fatigue and subjective fatigue perception. We regressed the increment of muscle fatigue against the increment of subjective fatigue perception for each group. We performed bootstrapping method to compare the regression coefficients of muscle fatigue changes among the three groups of two studies respectively. As shown in Figure 7C, advanced and delayed groups of both modalities showed difference with the standard group for both modalities. And the interaction between the sensory prediction error and muscle fatigue is regardless of the direction of sensory prediction errors for both modalities.

Taken together, consistent with our hypothesis, the sensory prediction error in the both modalities during exercise relates with fluctuations of subjective fatigue perception. Meanwhile, the fatigue of the main muscles exerting force locally is also involved in this process and has an interactive effect with sensory prediction error.

### Computational models

When establishing the models, standardized data were used. After standardization, temporal errors (TEs) were not zero and could be regarded as a constant, but spatial errors (SEs) in the standard group were zero. Therefore, we did not use the hyperbolic form of SE to establish the spatial modality models. The AIC and BIC values for each model in each group can be found in supplemental information.

Model group 1: the input of model group 1 is the sensory error introduced in the experiment, which is used to fit the fluctuations of subjective fatigue perception. In temporal modality, the model form with the best fitting effect is:

$$SF=aTE^{2}+b$$

In spatial modality, the model form with the best fitting effect is:

$$SF=aSE^{2}+b$$

The trend conforms to the predictive coding theory, that as sensory prediction error increases, the rate of change of subjective fatigue perception gradually increases and is independent of the direction of error.

Model group 2: the input of model group 2 is the muscle fatigue characteristics collected in the experiment. In temporal modality, the model form with the best fitting effect is

SF=a/MF+b

In spatial modality, the model form with the best fitting effect is

$$SF=aMF^{2}+b$$

Model group 3: our experimental results also indicate that perceptual prediction errors and muscle fatigue are both related to subjective fatigue perception (Figure 6). We took the muscle fatigue and sensory prediction error as independent variables respectively. In temporal modality, the model form with the best fitting effect is

$$SF=aMF^{2}+bTE$$

In spatial modality, the model form with the best fitting effect is

$$SF=aMF^{2}+bSE^{2}$$

Different from temporal modality, the sensory prediction error does not have a directional effect.

Model group 4: model group 4 added the interaction into model group 3. In temporal modality, the model form with the best fitting effect is

$$SF=aTE\;\cdot \;MF^{2}+b/TE$$

In spatial modality, the model form with the best fitting effect is

$$SF=ae^{SE}MF^{2}+bSE^{2}$$

In the second term, the amplitude of the SF’s fluctuations is inversely proportional to the magnitude of TE, and the direction is related to the positive or negative value of TE. The impact of SE on the fluctuations of subjective fatigue perception still does not have a directional effect.

Model group 5: in temporal modality, the model form with the best fitting effect is

$$SF=aTE^{2}e^{MF}+bTE+cHR$$

In spatial modality, the model form with the best fitting effect is

$$SF=a\cdot e^{SE}MF+bSE+c/HR$$

**Figure 8:**
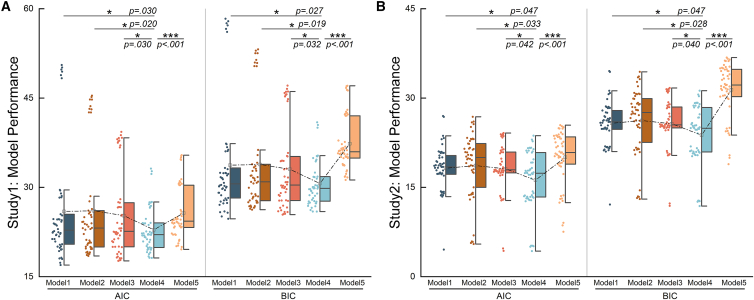
Comparison of the performance of the optimal models among different model groups in spatiotemporal modality (A) Results of temporal modality. (AIC: model1 vs. model4: DF = 96, *p* = 0.030, Cohen’s d = 0.45, 95%CI = [0.41, 6.11], model2 vs. model4: DF = 96, *p* = 0.020, Cohen’s d = 0.48, 95%CI = [0.62, 5.99], model3 vs. model4: DF = 96, *p* = 0.030, Cohen’s d = 0.44, 95%CI = [0.28, 4.87], model5 vs. model4: DF = 96, *p* < 0.001, Cohen’s d = 0.69, 95%CI = [1.26, 4.52], BIC: model1 vs. model4: DF = 96, *p* = 0.027, Cohen’s d = 0.45, 95%CI = [0.56, 6.11], model2 vs. model4: DF = 96, *p* = 0.019, Cohen’s d = 0.48, 95%CI = [0.49, 5.90], model3 vs. model4: DF = 96, *p* = 0.032, Cohen’s d = 0.44, 95%CI = [0.15, 4.77], model5 vs. model4: DF = 96, *p* < 0.001, Cohen’s d = 1.64, 95%CI = [5.08, 8.38]). (B) Results of spatial modality. Both study results indicate that model group4 performs the best. (AIC: model1 vs. model4: DF = 96, *p* = 0.047, Cohen’s d = 0.41, 95%CI = [0.14, 3.48], model2 vs. model4: DF = 96, *p* = 0.033, Cohen’s d = 0.44, 95%CI = [0.13, 4.33], model3 vs. model4: DF = 96, *p* = 0.042, Cohen’s d = 0.42, 95%CI = [0.15, 3.84], model5 vs. model4: DF = 96, *p* < 0.001, Cohen’s d = 0.82, 95%CI = [2.05, 5.77], BIC: model1 vs. model4: DF = 96, *p* = 0.047, Cohen’s d = 0.41, 95%CI = [0.07, 3.70], model2 vs. model4: DF = 96, *p* = 0.028, Cohen’s d = 0.44, 95%CI = [0.42, 4.29], model3 vs. model4: DF = 96, *p* = 0.040, Cohen’s d = 0.42, 95%CI = [0.16, 3.78], model5 vs. model4: DF = 96, *p* < 0.001, Cohen’s d = 1.61, 95%CI = [5.81, 9.60]).

To find the optimal model among the above model groups, we compared the fitting performance across the best model within each model group. The results showed that the optimal model in model group 4 exhibited the best model performance (Figure 8) whether in the temporal modality or in the spatial modality. According to Occam’s razor principle, we can consider that the computational model form of model group 4 can best fit the process in which the artificially introduced sensory prediction error, muscle fatigue and their interaction associate with the subjective fatigue perception, which fits our analysis results in the second section of results and our hypotheses.

### Pooled analysis

In the following, we answer the question whether there are common rules across different modalities. We performed the pooled analysis by putting the data of both modalities together into a regression model in which the experimental condition, the change value of the muscle fatigue state, the modality (temporal modality = 1, spatial modality = 2), and various interactions were used as independent variables to regress against the changes in subjective fatigue perception. The main effects of the experimental condition (*p* =0.048, estimate = −2.861) and the change value of the muscle fatigue state (*p* = 0.048, estimate = 0.085) were significant. We also found a significant interaction between the experimental condition and the changes of the muscle fatigue state (*p* = 0.005, estimate = −0.036). The remaining effect and interactions were not significant. This suggests that the relationship among subjective fatigue perception, muscle fatigue state and sensory prediction errors depends on whether the error was advanced or delayed in nature, which is common across modalities.

### Robustness analysis using a composite fatigue index

We calculated the average fatigue index (FI) 24 value for each participant in each block. And re-running our comparison and computational modeling pipeline with this metric yielded results that were fully consistent with our primary findings (Figure S1 in supplementary information1). The optimal model (model group 4) retained the best fit, and the modality-specific interaction patterns between sensory prediction error and muscle fatigue remained identical. This confirms that the identified computational mechanism of fatigue perception is robust to the specific electrophysiological representation of local muscle fatigue.

Furthermore, to verify the sensory distortion that may be caused by muscle fatigue 25 and to explain the different interaction patterns of errors in the two modalities compared to muscle fatigue, we quantified sensory distortion as the absolute error between the participant’s subjective estimate of the total exercise duration/distance and the actual value. We then establish a regression model, with this distortion as the dependent variable, the FI as the independent variable, and the group (standard group = 1, advanced group = 2 and delayed group = 3) as the covariate. We found a positive relationship between muscle fatigue and sensory distortion in both modalities (temporal modality: estimate = 0.174, *p* = 0.010, spatial modality: estimate = 0.167, *p* = 0.027), indicating that participants with higher levels of muscle fatigue were significantly worse at accurately judging the duration/distance they had run.

## Discussion

Brain and body constitute the whole human, indicating the close interaction between cognitive and physical domains. The fatigue induced by prolonged exercise intersects between both domains, which, however, has been largely studied separately within either cognitive or physical domain.^24^^,^^25^^,^^26^^,^^27^ In the present study, we investigated how local muscle fatigue and prediction error associate with fatigue perception, aiming to fill in the gap of how physical and cognitive aspects interact with each other and associate with subjective fatigue perception. We utilized the running task, a natural protocol close to daily exercise scenarios, and introduced sensory prediction errors with two temporal and spatial modalities. We showed that not only sensory prediction errors relate with subjective fatigue perception, as indicated by previous studies,^28^^,^^29^ but muscle fatigue interacting with sensory prediction errors also present significant correlation with the fluctuation of subjective fatigue perception. By using computational modeling, we further revealed that the specific association between muscle fatigue, sensory prediction errors and subjective fatigue perception differed across modalities, suggesting a modality-dependent computational mechanism underlying the dynamics of subjective fatigue perception during exercise. These results highlight that the subjective fatigue induced by prolonged exercise (1) fluctuate due to both sensory prediction error in the cognitive domain and local muscle fatigue in the physical domain and (2) is sensitive to the modality of sensory prediction error in terms of underlying computational mechanism.

Our study presents a novel and generalizable computational and analyzing framework that may aim future studies. We combined associative analysis and computation modeling to first investigate the relationship between independent variables, which in our case are sensory prediction error, muscle fatigue, heart rate, and pleasure levels, and dependent variables. We got the associative prior identifying the independent variables with significance that helps to select the input for computational models. By computationally modeling the associations, we further reveal the computational process of the form in which each independent variable associates with dependent variable and find the difference across modalities. The computational models selected by fitting performance, which in other words account the best for the observations, suggest the computational mechanism of human body underlying the observed phenomena where just some mental or physiological features vary together. This could be used in future studies to further test the association between the computational model-estimated parameters with neuroimages and to determine the neural correlates of the behavioral observations. Taken together, associative analysis indicates the significant associations between independent and dependent variables to screen input variables for computational modeling. Computation models confirm the findings of associative studies and further reveal more specific computational mechanism underlying associations. Although similar framework can be found in effort-related decision making,^30^^,^^31^^,^^32^ to our knowledge, our proposed framework is the first one applied on brain-body interaction that measured mental and physiological states together.

The findings of our study may contribute to the literature in the following aspects. First, compared with previous studies using hand grasp, isometric contraction as experimental protocol to induce fatigue, we used running, a more natural protocol close to daily exercise. As indicated,^33^^,^^34^^,^^35^ a natural experimental protocol may provide higher ecological validity, as it mimics real-world physical activities and engages integrated physiological systems (e.g., cardiovascular, metabolic, and neuromuscular systems), thereby reflecting holistic cognitive and physical fatigue processes. Also, running inherently involves central-peripheral integration, where afferent signals from fatigued muscles modulate cortical motor output-a phenomenon less pronounced in isolated muscle tasks.^36^ Findings from running paradigms directly inform practical applications, such as optimizing endurance training or designing fatigue-mitigation strategies in sports and occupational settings, bridging the gap between laboratory research and real-world scenarios.^37^^,^^38^ Second, our study provided a unified framework to understand how the cognitive and physical aspects of human associate with the fatigue perception. Classic fatigue perception theory considers fatigue perception as a result of the accumulation of effort feelings, which mainly associate with potential reward and the mismatch between motor prediction and actual performance. Despite sparse evidence, previous studies focused locally on how the cognitive aspects (e.g., sensory prediction error,^29^ reward,^39^ decision,^40^ etc.) or on the physical aspects (e.g., motor performance,^41^ breathing,^42^ muscle states,^43^ etc.) associate with fatigue perception. Although review and perspective papers^4^^,^^20^^,^^44^ pointed out the view of brain-body interaction during the generation of fatigue perception. Brain-body interaction is a multifaceted and dynamic process, which involves the integration of physiological signals from the body,^45^ the regulation of cognitive factors in the brain,^5^^,^^46^ as well as the influence of neural plasticity.^47^ Experimental observations that cover the bodily states and sensory manipulation is still insufficient to fill in the gap of how both domains interact and associate with fluctuations of fatigue perception. We tried to exclude the influence of reward on fatigue perception, given that reward is crucial factor within decision-making circuits.^48^^,^^49^ Our results provided the experimental observations and supported the integrative view by both data statistical analysis and computational modeling, which confirmed each other and thus strengthened the evidence. This suggests the intrinsic bidirectional interactions between muscle and brain, similar findings of which have been shown across peripheral systems, including breathing,^50^ muscle,^51^ stomach, and gut.^52^^,^^53^

Third, on sensory prediction error, previous studies^5^ that manipulated motor performance and prediction in a spatiotemporal coupling manner. That is, the sensor prediction errors were induced by changing both time and distance of performing a motor task. However, the processing mechanism of sensory cortex presented difference between temporal and spatial perception,^54^^,^^55^ which suggests different mechanisms underlying sensory prediction errors across modalities and thus may induce different impacts on fatigue perception. Although our results of pooled analyses showed that sensory prediction error in both modalities, as well as their interaction with muscle fatigue, presented significant correlation with fatigue perception, our computational model showed that the mathematical forms of sensory prediction errors and their interaction with muscle fatigue were largely different across modalities. This suggests the different mechanism of processing spatial and temporal sensory feedback when processing fatigue perception. We found a correlation between sensory distortion and muscle fatigue. This corruption likely forces the brain to rely on increasingly uncertain predictions,^56^^,^^57^ and the weight of muscle fatigue in spatial modality is greater than that in temporal modality, explaining the exponential amplification we observed when spatial prediction errors interact with muscle fatigue while the temporal modality appears more resilient to this effect, consistent with our linear interaction term.

Finally, adding heart rate into computational models did not significantly improve the fitting performance, despite the associative analysis showed heart rate and its interaction with conditions or muscle fatigue associated with subjective fatigue perception within temporal modality, also heart rate-condition, heart rate-muscle fatigue interaction terms associated with subjective fatigue perception within spatial modality. This suggests a broad brain-body interaction during the fluctuation of subjective fatigue perception, which involves in our case cardiac and muscular systems. The variations of both systems may reflect partial perspectives of the bodily states, are sensed and then regulated by the brain, thus associate with subjective fatigue perception.

While our study focused on sensorimotor mechanisms of fatigue, we acknowledge that cognitive and psychological factors (e.g., sleep, stress, emotion) also influence fatigue perception.^58^ These factors vary substantially between individuals and may explain baseline differences in fatigue susceptibility.^59^ So, our within-subject design and modeling of relative changes from individual baselines ensure that our core findings are robust to such stable individual differences. In this way, we believe the difference of fatigue feelings across groups are generally the effect of sensorimotor mismatch and local muscle fatigue. Future research could incorporate broader cognitive and lifestyle factors or integrate data such as electroencephalography to identify emotional states^60^ to explore how they tune the fatigue experience.

### Potential neural mechanism underlying the interplay between sensory prediction error and local muscle fatigue

The potential neural mechanisms underlying the sensory prediction error-bodily state interactive association with fatigue perception could be due to the intersection between interoceptive and sensorimotor systems. In the context of fatigue, sensory prediction error may play a crucial role in how the brain perceives and responds to the body’s changing state.^5^ Sensory prediction error is mainly mediated by the cerebellum-thalamus-cortex loop and the basal ganglia circuit.^61^^,^^62^ The cerebellum predicts the motor outcome through a forward model, while the dorsal anterior cingulate cortex (dACC) and the supplementary motor area (SMA) encode the prediction error by comparing the model with interoceptive signals.^47^^,^^63^^,^^64^^,^^65^ It is worth noting that the intensity of the sensory prediction error is related to the activation level of the dACC,^66^ which affects the perception of subjective effort.^67^ Subjective fatigue may be regulated by the anterior insular cortex (AIC)-dACC axis, which integrates interoceptive signals and assigns emotional salience to physical exertion.^68^^,^^69^^,^^70^ The ventromedial prefrontal cortex and the posterior cingulate cortex (PCC) further regulate the perception of the psychological state through the self-referential processing of the default mode network (DMN).^71^^,^^72^ The AIC, dACC, and posterior mid-cingulate cortex form the core hub of interoception, receiving interoceptive inputs through neural afferents.^73^ This network dynamically updates the brain’s perception of the body state and affects fatigue-related decisions.^74^

As key convergence nodes, the AIC and dACC are where interoceptive signals may interact with the calculation of sensory prediction errors.^69^ Neuroimaging evidence shows that the communication between the AIC and the motor cortex is enhanced during long-term exercise.^75^ The activation of the AIC is not only related to the intensity of the sensory prediction error,^76^ but also related to the self-reported psychological feeling scores.^77^ These errors can then be integrated with interoceptive signals related to the physical fatigue state, further regulating the subjective experience of fatigue. The salience network (SN) with the AIC and dACC as the core coordinates the dynamic switching between the DMN and the frontoparietal control network.^78^^,^^79^ During the accumulation of exercise, the SN may amplify the perception of fatigue by enhancing the salience of sensory prediction errors and interoception.

Taken together, the neural mechanisms underlying the interactive association among sensory prediction error, body state, and fatigue perception are complex and involve the coordinated activities of multiple brain regions and neural circuits.^80^^,^^81^ The intersection of the interoceptive system and the sensorimotor system, as well as the roles of key brain regions such as the AIC, dACC, and PFC, may provide a framework for understanding how the brain processes and responds to the body’s changing state during physical activities and the subsequent experience of fatigue. Future research can focus on further clarifying the specific neural mechanisms involved in this interaction to develop more targeted interventions for managing fatigue.

In conclusion, fatigue perception during exercise involves dynamic interactions between physical and cognitive factors. By combining the associative analysis and computational modeling, this study quantitatively reveals from the natural task that both muscle fatigue and spatiotemporal sensory prediction errors associate with subjective fatigue, with modality-specific interactions: temporal errors linearly amplify muscle fatigue’s effect, while spatial errors exhibit exponential modulation. These findings challenge the classical fatigue perception model solely rendering on cognitive aspects by further showing the modality-specific effects of muscle fatigue. The study provides a unified computational framework for brain-body interactions in fatigue, offering insights for personalized training and interventions targeting both physical and cognitive pathways.

### Limitations of the study

This study was conducted among healthy young adults, which may limit the applicability of the research results to other age groups. Additionally, future studies could collect neuroimaging data to validate the neural mechanisms proposed in this study.

## Resource availability

### Lead contact

Further information and requests for resources should be directed to and will be fulfilled by the lead contact, Chunzhi Yi (chunzhiyi@hit.edu.cn).

### Materials availability

This study did not generate any relevant materials.

### Data and code availability

• All data generated in this study are included in the article.
• Analysis codes presented in the text are also available on OSF: https://osf.io/pycxr/?view_only=d3ad2737b8414e208ffef0d06f6ae548.
• Any additional information required to reanalyze the data reported in this study is available from the lead contact upon request.

## Acknowledgments

This work was supported by the 10.13039/501100012166National Key Research and Development Program of China (no. 2024YFC3016403), 10.13039/501100001809National Natural Science Foundation of China (no. 62306083), and in part by the 10.13039/501100006579Ministry of Industry and Information Technology of China.

## Author contributions

Conceptualization, C.Y., Z.C., C.Y., and H.Z.; methodology, Z.X. and C.Y.; experiment, Z.X., C.Z., C.Y., and B.W.; writing – original draft, Z.X. and C.Y.; writing – review and editing, C.Y. and S.C.

## Declaration of interests

The authors declare no competing interests.

## STAR★Methods

### Key resources table

**Table:** 

REAGENT or RESOURCE	SOURCE	IDENTIFIER
**Deposited data**		

Experimental Data and Codes	This paper	OSF: https://osf.io/pycxr/?view_only=d3ad2737b8414e208ffef0d06f6ae548
Surface electromyogram: Wireless Surface EMG Sensors: Trigno Avanti	DELSYS, USA	N/A
Synchronizing signal: Trigger Module	DELSYS, USA	N/A
Heart Rate: Monitoring Smart Bracelet: WS20A	Accbiomed, China	N/A
Foot sole pressure: Pressure Sensors: FSR402	Interlink Electronics, USA	N/A
NI myRIO 1900	National Instruments, USA	N/A

**Software and algorithms**		

MATLAB 2022b	MathWorks, USA	https://www.mathworks.com/
Levenberg-Marquardt Algorithm Implementation	MATLAB Optimization Toolbox	https://ww2.mathworks.cn/products/optimization.html
Fourth-order Butterworth Band-pass Filter (20–500 Hz)	MATLAB Signal Processing Toolbox	https://ww2.mathworks.cn/products/signal.html
50 Hz Notch Filter	MATLAB Signal Processing Toolbox	https://ww2.mathworks.cn/products/signal.html
Principal Component Analysis (PCA)	MATLAB Statistics and Machine Learning Toolbox	https://ww2.mathworks.cn/products/statistics.html
Permutation Test Statistical Analysis	This paper and OSF Repository	Described in method details and codes in OSF: https://osf.io/pycxr/?view_only=d3ad2737b8414e208ffef0d06f6ae548
Origin 2024	OriginLab	https://www.originlab.com

### Experimental model and study participant details

We recruited 57 healthy young participants across two studies. Study 1 investigated the effect of temporal prediction error on physical fatigue (*n* = 30 participants, 17 females, 13 males, age range 18–27). Study 2 investigated the effect of spatial prediction error on physical fatigue (*n* = 27 participants, 13 females, 14 males). The potential effects of sex were not investigated in the present study, which may be a limitation to generalizability. Both studies were approved by the Chinese Ethics Committee of Registering Clinical Trials (ChiECRCT20200319). Informed consent was obtained from all participants, including a health status declaration. We strictly required the participants to ensure their sleep quality and avoid consuming substances such as alcohol and caffeine and received an oral report from each participant on their current state before every experiment. For both studies, participants were compensated a flat rate of $5 for their time.

### Method details

#### Design

The difference between the two studies was whether the prediction error was temporal or spatial. Each study consisted of three phases: calibration phase, training phase and main task phase. The main task of each study was divided into three groups: standard, advanced and delayed groups, with each participant completing all the three groups in a random order to reduce the influence of individual differences and order effect. During the experiment, we also collected participants' subjective pleasure perception levels through asking ‘how to rate your pleasure?’ in Likert-scale questionnaires before and after the experiment apart from fatigue perception. This was to exclude the potential influence of exercise-related pleasure that may affect the progression of fatigue perception. A 7-point Likert scale was employed for subjective ratings to minimize cognitive load during the physically demanding task and to maintain consistency in measuring both fatigue and pleasure. The calibration phase aimed at establishing a baseline for participants' fatigue perception levels and setting an appropriate speed for the main task. And the training phase was to familiarize participants with the experimental procedure including alert sounds and ambient sounds (Figure 1). During all stages of the experiment, we turned off non-essential electronic devices and used headphones to play the sounds to minimize the interference from environmental noises to the greatest extent. And there was a one-week interval between each experiment to ensure that the participants' fatigue had completely recovered.

#### Calibration

The calibration phase aimed to familiarize the participants with the feelings corresponding to different fatigue perception levels. The participants were required to increase their running speed to the greatest extent until they could not maintain a stable motion state for 30 s, during which strong verbal encouragement was provided. The speed at this moment was defined as the MRV that the participant could tolerate. To normalize fatigue perception levels across participants and avoid variability due to differences in physical condition, we told the fatigue perception of maintaining MRV for 30 s corresponded to fatigue level 4. There were totally 7 fatigue levels (Figure 1B). The treadmill’s speed was set at 70% MRV in the main task. Subsequently, the subjects took a rest until they verbally reported that they were no longer fatigued, and then returned to the treadmill for the following phases.

#### Training

In both studies, each participant completed an initial training phase before their first participation in the main task. In the first part of the training phase, participants were familiarized with the treadmill and informed that during the formal experiment, they should naturally look straight ahead, focusing their gaze on the central part of the cross on the screen in front of them to avoid the interference of other visual feedback.

In the second part of the training phase, the experimenters introduced the procedure to the participants, set phased exercise goals, and explained the way to report the fatigue level and the pleasure level. We informed the achievement of the participants per block. Specifically, in the study of the temporal modality, the participants ran for 20 min, consisting of 4 blocks. During each block, the participants ran for 5 min. Upon completing a block, they were verbally informed by the experimenter that 'You have run for 5 min'. In the study of the spatial modality, the participants ran for 3 km, consisting of 4 blocks. During each block, the participants ran for 750 m. Upon completing a block, they were verbally informed by the experimenter that 'You have run for 750 m' (Figure 1B). Upon hearing information, the participants were required to verbally report their current fatigue perception level and pleasure perception level within 5 s. The experimenters recorded the current levels of fatigue and pleasure perception. In the last block, the participants were asked to answer a question about the perceived exercise time or distance. A training of 10 min or 1500 m was respectively conducted for the temporal and spatial modalities to ensure that the participants were familiar with the experimental procedure and can answer the corresponding questions.

#### Main task

This was the main phase of the experiment during which most primary outcome measures related to fatigue were taken. In the main task, both studies consisted of 4 blocks (Figure 1C). For each block, all the participants were verbally informed that they would run 5 min per block for Study 1 and 750 m per block for Study 2. Different from the training phase, the participants were unaware of their actual running time or distance, which varied across the standard, delayed and advanced groups, to induce the prediction errors. Specifically, in the standard group, participants were verbally informed the actual time or distance. In the advanced group, verbal information was provided before the participants actually achieved the informed running time or distance. In the delayed group, verbal information was provided later than the participants actually achieved the informed target. The verbal information was pre-generated by computer.

#### Temporal task

In the standard group, the actual running time for the whole session was 20 min, each block lasting 5 min. The participants were verbally informed that ‘You have already run for 5 min’ at the end of each block. Then, participants were asked to rate from 1 to 7 of ‘how fatigued you felt’ and ‘how pleased you felt by running’ immediately. In the advanced group, the total actual running time was 15 min. The actual running time for each block was sampled without replacement from the set {4.5 min, 4.0 min, 3.5 min, 3.0 min} for each subject. In the delayed group, the total actual running time was 25 min. The actual running time was sampled from the set {5.5 min, 6.0 min, 6.5 min, 7.0 min}. The participants were informed with the same content and asked the same questions as the standard group in the other two groups. During the final block, the participants were asked ‘How long do you feel you have been running?’ to inquire about the perceived running time.

#### Spatial task

Similarly, the participants were verbally informed that ‘You have already run for 750 m’ and the same questions for the fatigue and pleasure levels were asked at the end of each block. The actual running distance for the whole session in the standard group was 3 km, each block lasting 750 m. In the advanced group, the total actual running distance was 2.25 km. The actual running distance for each block was sampled from the set {675 m, 600 m, 525 m, 450. In the delayed group, the total actual running distance was 3.75 km. The actual running distance was sampled from the set {825 m, 900 m, 975 m, 1050 m}. During the final block, the participants were asked ‘How far do you feel you have been running?’ to inquire about the perceived running distance.

#### Apparatus

Wireless surface electromyography (EMG) sensors (Trigno Avanti, DELSYS, USA, 1111 Hz) were used to measure the electrical signals on the surface of the participants' muscles. The EMG sensors were placed on the right rectus femoris and the anterior tibialis.^82^ Heart rate was monitored by a smart bracelet (WS20A, Accbiomed, China, 10 Hz), and the data was received in real time via Bluetooth by a laptop. The bracelet was worn on the left wrist of every participant (Figure 1A). Pressure sensors (FSR402, 100 Hz) were attaced to the heel and first metatarsal bone of the left and right feet, respectively, the signals of which were collected by the embeded microprocessor (NI myRIO 1900). Signals synchronization was achieved by the Trigger Module (DELSYS, USA). The screen of the treadmill was coverd and the sound was muted in order to refrain the participants from knowing the actual running time or distance. The verbal information in the experiment was played through a Bluetooth audio player. Data processing and statistical analysis were programmed using MATLAB 2022b (MathWorks, USA).

#### Data processing and statistical analysis

The EMG data was collected at a sampling rate of 1111 Hz. During the subsequent analysis, a notch filter with a cutoff frequency of 50 Hz was utilized to eliminate the power frequency noise. Then, the EMG signal was filtered using a fourth-order Butterworth band-pass filter between 20 and 500 Hz. The EMG signal of each step was segmented using gait information, and the filtered data of each muscle was quantified as the median frequency (MF) of each step, used to characterize the muscle fatigue state.^83^^,^^84^ We also checked the signal quality of each segmented fragment one by one, and removed the EMG signals that clearly did not belong to regular movements. We used heart rate as a global indicator for peripheral fatigue^85^ in order to include peripheral indicators except for EMG of local muscles, which may also contribute to the variation of subjective fatigue feelings. First, we checked the heart rate signal for any outliers caused by the shaking of the fitness tracker bracelet. These unusual values might be due to measurement errors, improper usage, or other factors. The normal heart rate range is 40–200 beats per minute. Data points outside this range were marked as outliers and removed from the dataset. Then, a low-pass filter with a cutoff frequency of 10 Hz was used to remove the high-frequency noise from the heart rate data. Each block is evenly divided into 10 parts, and the average value of each part’s feature is taken as the indicator. The prediction error was calculated as the difference between actual running time (or distance) and the time (or distance) verbally told, with the negative error for the advanced group and the positive error for the delayed group.

Also, to address the potential limitation of relying solely on spectral characteristics, we performed a supplementary analysis using a composite Fatigue Index (FI)^56^ defined as

$$Fatigue\;Index=\frac{Amplitude/Amplitude_{baseline}}{Mean\;frequency/Mean\;frequency_{baseline}}$$

We calculated the average FI value for each participant in each block. And re-running our comparison and computational modeling pipeline.

#### Computational modeling

To investigate how peripheral fatigue and prediction errors of each modality interacted to form subjective fatigue perception, we developed computational models that predicted subjective fatigue perception from peripheral indicators and prediction errors in both modalities. On constructing the computational model, for each block, we averaged the MF and heart rate across the sampling points within the block, and calculated the differences from the baseline to eliminate the baseline variations. And we used the first principal component of MF of the two muscles from PCA to develop models. The sensory prediction error was defined as the unreal exercise duration/distance prompted to the subjects minus the real exercise duration/distance of the subjects. We z-scored all the variables including in the computational models. The model comparison would aim the understanding of the computational mechanism during the fluctuation of subjective fatigue perception.

We obtained the approximate mathematical function form for each variable in a step-by-step manner. First, for each independent variable, we used four common elementary function forms to fit the subjective fatigue perception levels respectively, the linear, the parabola, the hyperbola, and the exponential function. These functions captured the simplest typical patterns of each independent variables.^86^ Then, we selected one form with the best fitting performance for each independent variable. Then, a multivariate model was established by combining the optimal function forms of the independent variables. The combination of different independent variables and the interaction among the variables were tested by the fitting performance and selected accordingly. In this way, we remained the mathematical expression with the optimal fitting performance as the computational model accounting for the empirical measurements. The inputs and outputs of different computational model groups are shown below.

**Table:** The independent and dependent variables of the model

Forms of independent variables	Model Group 1 independent variables	Model Group 2 independent variables	Model Group 3 independent variables	Model Group 4 independent variables	Model Group 5 independent variables
Linear Parabola Hyperbola Exponent	Temporal Error (TE) Or Spatial Error (SE)	Muscle Fatigue (MF)	MF TE/SE	MF TE/SE MF × TE/SE	MF TE/SE MF × TE/SE Heart Rate (HR)
Model output Dependent variable	Subjective Fatigue (SF)				

##### Model Group 1: Prediction error model

According to previous studies,^28^^,^^29^ during the exercise process, due to the artificially introduced sensory prediction error, the actual feedback received by the human body may not match the efference copy generated simultaneously with the motor commands issued by the brain, resulting in intensified sensations, among which subjective fatigue perception may be included. Therefore, we developed a model with only the sensory feedback error terms as the independent variable for the temporal and spatial modalities, respectively, given by

$$SF_{i}=f\left(TE_{i}\right)$$

$$SF_{i}=f\left(SE_{i}\right)$$

where *SF*_*i*_ represents the subjective fatigue perception level of the i-th block.

##### Model Group 2: Muscle fatigue model

The afferent feedback of muscles and the muscle fatigue-induced adaptation of sensorimotor cortex^87^^,^^88^^,^^89^ suggested the possible contribution of muscle fatigue to the subjective fatigue perception. Also, our daily experience of exercising also suggested the association between soreness of muscles and fatigue feelings. Herein, we developed a model with only the muscle fatigue characteristic as the independent variable. This model did not distinguish across temporal and spatial modalities, given that we only evaluated in which function form MF should be included into following modeling. The model can be denoted by

$$SF_{i}=f\left(MF_{i}\right)$$

##### Model 3: Muscle fatigue and prediction error model

In this model, we treated muscle fatigue and prediction error as separate independent variables without considering their interactions, denoted by

$$SF_{i}=f\left(MF_{i},TE_{i}\right)$$

$$SF_{i}=f\left(MF_{i},SE_{i}\right)$$

Previous studies have shown that in simple finger movements, the sensory feedback error in the temporal modality introduced by adjusting the time interval of visual feedback can affect subjective fatigue perception, and the error has a directional effect.^5^ Therefore, *TE*_*i*_ and *SE*_*i*_ were with directions (negative, positive or equal to error).

##### Model 4: Muscle fatigue and prediction error interacting model

Previous study on the isometric contraction task of the thigh has shown the possibility that the fluctuation of muscle fatigue may relate with prediction error.^22^ In this model, we further included the potential interaction between prediction error and muscle fatigue by considering the interaction term of the prediction error and muscle fatigue, given by

$$SF_{i}=f\left(MF_{i}\times TE_{i},TE_{i}\right)$$

$$SF_{i}=f\left(MF_{i}\times SE_{i},SE_{i}\right)$$

##### Model 5: Heart rate-involved model

The changes in heart rate during exercise can reflect the global peripheral fatigue of the human body, such as the state of the sympathetic nervous system.^90^ To explore whether the changes in the global peripheral fatigue were involved in the fluctuation of subjective fatigue perception, after comparing the performance of the above models, heart rate was added as a separate independent variable into each of the above models with the best performance, given by

$$SF_{i}=ModelGroup_{1-4}+f\left(HR_{i}\right)$$

#### Model fitting

To fit the above models to the subjective fatigue levels of each stage during the exercise process $\tilde{SF}\left(i\right)$, we used the Levenberg-Marquardt algorithm to minimize an error *ERR*_*SF*_ defined by the sum of squared residuals between the subjective fatigue levels of each stage and the subjective fatigue perception estimated by the model *SF*(*i*):

$$ERR_{SF}=\sum_{i}{\left(SF\left(i\right)-\tilde{SF}\left(i\right)\right)}^{2}$$

The subjective fatigue levels collected in the experiment were first normalized to account for variability in scale usage between participants using

$$\tilde{SF}\left(i\right)=\frac{SF\left(i\right)-avg\left(SF\left(i\right)\right)}{std\left(SF\left(i\right)\right)}$$

where *avg* and *std* are computing the average and standard deviation of each subject’s levels.

Each term in the model contains a parameter to be estimated. The MATLAB lsqnonlin non-linear least squares solving function is utilized to optimize each model parameter under the constraint that it should be a positive number. The initial parameter values are randomly set within the open interval (0,1). Each model is fitted 50 times while ensuring that the optimization function does not get trapped in local minima.

Our models focus on determining whether subjective fatigue perception is best described by a certain model. Meanwhile, more parsimonious models are preferred over more complex ones. We use the Akaike Information Criterion (AIC) and the Bayesian Information Criterion (BIC) as comparison indicators for model performance, which punishes models for their number of free parameters. Under the assumption that the errors follow a normal distribution, we transform *ERR*_*SF*_ into likelihood functions and obtain the following criteria:

$$AIC=2k+n*\ln\left(ERR_{SF}\right)$$

$$BIC=k\;\ln\left(n\right)+n*\ln\left(ERR_{SF}\right)$$

where *k* is the number of model parameters and *n* is the number of samples.

We conducted a comparative analysis among multiple groups of models to obtain the optimal computational models under different modalities: For the computational models of different modalities, we first determined the optimal model in each Model Group by identifying the single model with the minimum AIC and BIC values. Secondly, we compared the optimal model in different model groups to find out the model with the best performance as the model of the dynamic process of subjective fatigue perception under the corresponding modality.

### Quantification and statistical analysis

During the data preprocessing stage, the Interquartile Range (IQR) method is employed to identify and remove outliers from the variables. This study employed the permutation test for statistical analysis. Due to the small sample size and the nonnormal distribution of some data, the permutation test does not rely on distribution assumptions and can provide more robust statistical inference results. Besides, we used Wilcoxon Signed-Rank Test to compare the differences between the perceived exercise process and the actual process of each group, and paired sample t-tests to examine pre- and post-exercise changes in physiological and psychological indices for each group, with results detailed in Figures 2 and 3. When comparing the physiological indicators, each block was evenly divided into 10 parts, and the characteristics of these parts were all used for comparison. Partial regression and multivariable linear regression were conducted to explore the associations between independent variables (muscle fatigue, heart rate, sensory prediction error, pleasure level) and subjective fatigue perception, and bootstrapping was used to compare regression coefficients of muscle fatigue changes across experimental groups. All statistical details including exact *n* values (n represents the number of participants, with Study 1: *n* = 30, Study 2: *n* = 27), center measures (mean), dispersion, and statistical test results are fully reported in the Results section, corresponding figures and figure legends. Statistical significance was defined as *p* < 0.05 for all analyses. For all statistical analyses, a Post hoc analysis was conducted using G∗Power to calculate the power of the results, with all the power greater than 0.85. For study design, participants were randomly assigned to the order of experimental conditions to minimize order effects and all participants were included in the final analysis. All statistical analysis was implemented using MATLAB 2022b (MathWorks, USA).

## Footnotes

Supplemental information can be found online at https://doi.org/10.1016/j.isci.2026.115552.
