# Supplementary material for: Physical and cognitive contributions to fatigue perception: The interplay between local muscle fatigue and sensory prediction error
Source: iScience. 2026 Apr 1;29(5):115552. doi: 10.1016/j.isci.2026.115552 (PMC13098603; doi:10.1016/j.isci.2026.115552)
Supplement: Document S1. Figure S1, Tables S1–S8, Data S1, and S2 [file mmc1.pdf]

iScience, Volume *29*

Supplemental information

Physical and cognitive contributions to fatigue perception: The interplay between local muscle fatigue and sensory prediction error

Zihang Xu, Baichun Wei, Chifu Yang, Haiqi Zhu, Chunyu Zhang, Zhiyuan Chen, Shuqing Chen, and Chunzhi Yi

As mentioned in the results section of the main text, for the optimal computational model selection methods of Study 1 and Study 2, we respectively selected the independent variable forms with the best model performance within each model group. In supplementary information, we list the forms of all the models participating in the comparison from Model Groups 1 to 5, as well as mean values and standard deviations of the AIC and BIC. The items marked in red in the table are the model with the best performance.

Study 1 – Temporal modality

1. Model Group 1: Prediction error model.

The models form is

$$SF_i = f(TE_i)$$

where SF_i represents the Subjective Fatigue perception level of the i-th block and TE_i represents the temporal error in different blocks.

Table S1 The model performance of Model Group 1 in Study 1

Forms of model	$\overline{\text{AIC}}$	$\overline{\text{BIC}}$	SD
SF = aTE+b	30.70	38.47	12.90
SF = aTE^2+b	25.96	33.73	9.22
SF = a/TE+b	29.44	37.21	11.10
SF = a*exp(TE) + b	29.93	37.70	13.20

2. Model Group 2: Muscle fatigue model.

The models form is

$$SF_i = f(MF_i)$$

where MF_i represents the Muscle Fatigue state of the i-th block.

Table S2 The model performance of Model Group 2 in Study 1

Forms of model	$\overline{\text{AIC}}$	$\overline{\text{BIC}}$	SD
SF = aMF+b	29.38	37.15	11.02
SF = aMF^2+b	29.31	37.08	8.91
SF = a/MF+b	26.07	33.84	8.73
SF = a*exp(MF)+b	30.62	38.40	10.76

3. Model Group 3: Muscle fatigue and prediction error model.

The models form is

$$SF_i = f(MF_i, TE_i)$$

Table S3 The model performance of Model Group 3 in Study 1

Forms of model	$\overline{\text{AIC}}$	$\overline{\text{BIC}}$	SD
SF=aMF+bTE	28.20	35.98	7.81
SF=aMF+bTE^2	28.35	36.12	9.66
SF=aMF+b/TE	28.03	35.80	8.20
SF=aMF+b*exp(TE)	28.77	36.54	10.44
SF=aMF^2+bTE	25.26	33.01	6.93
SF=aMF^2+bTE^2	28.45	36.22	7.97
SF=aMF^2+b/TE	28.49	36.26	8.17
SF=aMF^2+b*exp(TE)	28.06	35.83	9.25
SF=a/MF+bTE	27.32	35.09	6.82
SF=a/MF+bTE^2	28.11	35.89	8.32
SF=a/MF+b/TE	27.87	35.64	9.80
SF=a/MF+b*exp(TE)	28.68	36.45	9.19
SF=a*exp(MF)+bTE	27.94	35.72	7.59
SF=a*exp(MF)+bTE^2	27.55	35.32	6.52
SF=a*exp(MF)+b/TE	27.99	35.77	9.21
SF=a*exp(MF)+b*exp(TE)	28.41	36.18	9.98

4. Model Group 4: Muscle fatigue and prediction error interacting model.

The models form is

$$SF_i = f(MF_i \times TE_i, TE_i)$$

Table S4 The model performance of Model Group 4 in Study 1

Forms of model	$\overline{\text{AIC}}$	$\overline{\text{BIC}}$	SD
SF=aMF*TE+bTE	25.08	32.85	5.85
SF=aMF*TE+bTE^2	25.69	33.47	6.09
SF=aMF*TE+b/TE	24.52	32.30	5.39
SF=aMF*TE+b*exp(TE)	24.42	32.20	5.40
SF=aMF*TE^2+bTE	25.58	33.36	7.28
SF=aMF*TE^2+bTE^2	24.74	32.52	6.59
SF=aMF*TE^2+b/TE	26.07	33.84	7.95
SF=aMF*TE^2+b*exp(TE)	27.09	34.86	9.36
SF=a(MF*TE)+bTE	25.15	32.92	6.10
SF=a(MF*TE)+bTE^2	25.22	32.99	6.40
SF=a(MF*TE)+b/TE	26.06	33.83	8.25
SF=a(MF*TE)+b*exp(TE)	26.02	33.79	8.40
SF=aMF*exp(TE)+bTE	24.74	32.51	6.06
SF=aMF*exp(TE)+bTE^2	25.57	33.34	7.41
SF=aMF*exp(TE)+b/TE	25.68	33.45	7.31
SF=aMF*exp(TE)+b*exp(TE)	26.44	34.21	7.40
SF=aMF^2*TE+bTE	24.98	32.75	5.60
SF=aMF^2*TE+bTE^2	26.31	34.08	7.76
SF=aMF^2*TE+b/TE	22.87	30.64	3.65
SF=aMF^2*TE+b*exp(TE)	26.57	34.34	8.51
SF=aMF^2*TE^2+bTE	24.39	32.16	5.13
SF=aMF^2*TE^2+bTE^2	25.17	32.95	5.52
SF=aMF^2*TE^2+b/TE	24.95	32.73	6.98
SF=aMF^2*TE^2+b*exp(TE)	25.39	33.17	7.12
SF=aMF^2/TE+bTE	25.76	33.53	7.56
SF=aMF^2/TE+bTE^2	25.30	33.08	7.20
SF=aMF^2/TE+b/TE	26.72	34.49	9.38
SF=aMF^2/TE+b*exp(TE)	28.03	35.80	9.13
SF=aMF^2*exp(TE)+bTE	26.20	33.97	7.65
SF=aMF^2*exp(TE)+bTE^2	25.55	33.32	6.27
SF=aMF^2*exp(TE)+b/TE	26.12	33.89	8.31
SF=aMF^2*exp(TE)+b*exp(TE)	25.09	32.87	5.77

Tab S4 The model performance of Model Group 4 in Study 1 (continued)

Forms of model	$\overline{AIC}$	$\overline{BIC}$	SD
SF=a/MF*TE+bTE	25.35	33.13	8.11
SF=a/MF*TE+bTE^2	26.76	34.53	11.77
SF=a/MF*TE+b/TE	26.91	34.67	12.45
SF=a/MF*TE+b*exp(TE)	26.18	33.95	11.08
SF=a/MF*TE^2+bTE	24.72	32.49	6.36
SF=a/MF*TE^2+bTE^2	25.13	32.90	5.65
SF=a/MF*TE^2+b/TE	25.51	33.28	6.99
SF=a/MF*TE^2+b*exp(TE)	26.25	34.02	7.82
SF=a/(MF*TE)+bTE	26.35	34.13	8.33
SF=a/(MF*TE)+bTE^2	25.36	33.13	7.27
SF=a/(MF*TE)+b/TE	25.44	33.21	8.40
SF=a/(MF*TE)+b*exp(TE)	26.13	33.91	8.70
SF=a/MF*exp(TE)+bTE	24.66	32.43	5.93
SF=a/MF*exp(TE)+bTE^2	25.01	32.78	6.03
SF=a/MF*exp(TE)+b/TE	24.72	32.50	4.87
SF=a/MF*exp(TE)+b*exp(TE)	27.28	35.05	7.28
SF=a*exp(MF)*TE+bTE	24.57	32.34	5.04
SF=a*exp(MF)*TE+bTE^2	24.56	32.33	4.87
SF=a*exp(MF)*TE+b/TE	28.70	36.47	10.16
SF=a*exp(MF)*TE+b*exp(TE)	24.66	32.43	6.06
SF=a*exp(MF)*TE^2+bTE	26.49	34.26	8.22
SF=a*exp(MF)*TE^2+bTE^2	24.15	31.92	3.68
SF=a*exp(MF)*TE^2+b/TE	24.47	32.25	5.56
SF=a*exp(MF)*TE^2+b*exp(TE)	24.47	32.24	4.95
SF=a*exp(MF)/TE+bTE	24.43	32.20	5.52
SF=a*exp(MF)/TE+bTE^2	25.30	33.08	7.04
SF=a*exp(MF)/TE+b/TE	25.00	32.78	7.52
SF=a*exp(MF)/TE+b*exp(TE)	26.68	34.45	8.90
SF=a*exp(MF)*exp(TE)+bTE	25.74	33.51	7.15
SF=a*exp(MF)*exp(TE)+bTE^2	25.09	32.87	5.91
SF=a*exp(MF)*exp(TE)+b/TE	25.80	33.58	7.76
SF=a*exp(MF)*exp(TE)+b*exp(TE)	25.51	33.28	7.94

5. **Model Group 5: Heart rate-involved model.**

The models form is

$$SF_i = ModelGroup_{1-4} + f(HR_i)$$

where HR_i represents the average of Heart Rate in the i-th block. The performance of each model can be found in Data S1.

Study 2 – Spatial modality

1. Model Group 1: Prediction error model.

The models form is

$$SF_i = f(SE_i)$$

where SF_i represents the Subjective Fatigue perception level of the i-th block and SE_i represents the spatial error in different blocks.

Table S5 The model performance of Model Group 1 in Study 2

Forms of model	$\overline{\text{AIC}}$	$\overline{\text{BIC}}$	SD
SF=aSE+b	20.25	28.02	3.88
SF=aSE^2+b	18.21	25.77	3.69
SF=a*exp(SE)+b	19.68	27.45	4.47

2. Model Group 2: Muscle fatigue model.

The models form is

$$SF_i = f(MF_i)$$

where MF_i represents the Muscle Fatigue state of the i-th block.

Table S6 The model performance of Model Group 2 in Study 2

Forms of model	$\overline{\text{AIC}}$	$\overline{\text{BIC}}$	SD
SF=aMF+b	20.32	28.09	3.57
SF=aMF.^2+b	18.66	26.22	5.49
SF=a/MF+b	22.07	29.84	12.09
SF=a*exp(MF)+b	20.52	28.29	3.94

3. Model Group 3: Muscle fatigue and prediction error model.

The models form is

$$SF_i = f(MF_i, SE_i)$$

Table S7 The model performance of Model Group 3 in Study 2

Forms of model	$\overline{\text{AIC}}$	$\overline{\text{BIC}}$	SD
SF=aMF+bSE	19.52	27.29	3.71
SF=aMF+bSE^2	19.83	27.6	3.59
SF=aMF+b*exp(SE)	19.77	27.54	5.31
SF=aMF^2+bSE	19.95	27.72	5.42
SF=aMF^2+bSE^2	18.11	25.67	4.20
SF=aMF^2+b*exp(SE)	19.82	27.59	4.89
SF=a/MF+bSE	21.20	28.97	12.71
SF=a/MF+bSE^2	22.61	30.38	14.81
SF=a/MF+b*exp(SE)	22.14	29.91	11.70
SF=a*exp(MF)+bSE	19.81	27.58	4.49
SF=a*exp(MF)+bSE^2	20.72	28.50	5.03
SF=a*exp(MF)+b*exp(SE)	19.50	27.27	4.05

4. Model Group 4: Muscle fatigue and prediction error interacting model.

The models form is

$$SF_i = f(MF_i \times SE_i, SE_i)$$

Table S8 The model performance of Model Group 4 in Study 2

Forms of model	$\overline{\text{AIC}}$	$\overline{\text{BIC}}$	SD
SF=aMF*SE+bSE	18.28	26.05	3.72
SF=aMF*SE+bSE^2	18.07	25.84	3.79
SF=aMF*SE+b*exp(SE)	18.59	26.36	3.44
SF=aMF*SE^2+bSE	18.12	25.89	4.51
SF=aMF*SE^2+bSE^2	18.11	25.88	4.40
SF=aMF*SE^2+b*exp(SE)	18.32	26.09	4.22
SF=aMF/SE+bSE	18.33	26.11	4.25
SF=aMF/SE+bSE^2	19.57	27.34	4.21
SF=aMF/SE+b*exp(SE)	20.02	27.79	4.82
SF=aMF*exp(SE)+bSE	18.87	26.64	5.03
SF=aMF*exp(SE)+bSE^2	17.85	25.63	4.67
SF=aMF*exp(SE)+b*exp(SE)	19.38	27.16	3.28
SF=aMF^2*SE+bSE	18.57	26.34	4.84
SF=aMF^2*SE+bSE^2	18.14	25.91	4.85
SF=aMF^2*SE+b*exp(SE)	19.44	27.21	4.10
SF=aMF^2*SE^2+bSE	19.17	26.94	5.11
SF=aMF^2*SE^2+bSE^2	19.40	27.17	6.26
SF=aMF^2*SE^2+b*exp(SE)	17.99	25.76	4.50
SF=aMF^2/SE+bSE	18.30	26.07	4.30
SF=aMF^2*/SE+bSE^2	19.23	27.01	3.51
SF=aMF^2/SE+b*exp(SE)	18.59	26.36	4.91
SF=aMF^2*exp(SE)+bSE	18.56	26.33	5.07
SF=aMF^2*exp(SE)+bSE^2	16.21	23.77	4.94
SF=aMF^2*exp(SE)+b*exp(SE)	18.00	25.78	5.03
SF=a/MF*SE+bSE	19.26	27.03	8.18
SF=a/MF*SE+bSE^2	19.17	26.94	6.25
SF=a/MF*SE+b*exp(SE)	20.40	28.17	8.25
SF=a/MF*SE^2+bSE	21.67	29.44	11.55
SF=a/MF*SE^2+bSE^2	20.42	28.20	8.31
SF=a/MF*SE^2+b*exp(SE)	20.26	28.03	8.82
SF=a/MF*exp(SE)+bSE	19.61	27.38	9.80
SF=a/MF*exp(SE)+bSE^2	20.54	28.31	9.85

Table S8 The model performance of Model Group 4 in Study 2 (continued)

Forms of model	$\overline{AIC}$	$\overline{BIC}$	SD
SF=a/MF*exp(SE)+b*exp(SE)	20.17	27.94	10.53
SF=a*exp(MF)*SE+bSE	18.42	26.20	3.78
SF=a*exp(MF)*SE+bSE^2	19.14	26.91	3.05
SF=a*exp(MF)*SE+b*exp(SE)	17.90	25.67	4.37
SF=a*exp(MF)*SE^2+bSE	19.18	26.95	3.86
SF=a*exp(MF)*SE^2+bSE^2	18.87	26.64	5.08
SF=a*exp(MF)*SE^2+b*exp(SE)	18.77	26.54	5.55
SF=a*exp(MF)/SE+bSE	18.55	26.33	4.54
SF=a*exp(MF)/SE+bSE^2	19.46	27.24	3.65
SF=a*exp(MF)/SE+b*exp(SE)	19.59	27.37	3.60
SF=a*exp(MF)*exp(SE)+bSE	18.69	26.47	5.88
SF=a*exp(MF)*exp(SE)+bSE^2	18.38	26.15	5.31
SF=a*exp(MF)*exp(SE)+b*exp(SE)	18.65	26.42	4.65

5. Model Group 5: Heart rate-involved model.

The models form is

$$SF_i = ModelGroup_{1-4} + f(HR_i)$$

where HR_i represents the average of Heart Rate in the i-th block. The performance of each model can be found in Data S2.

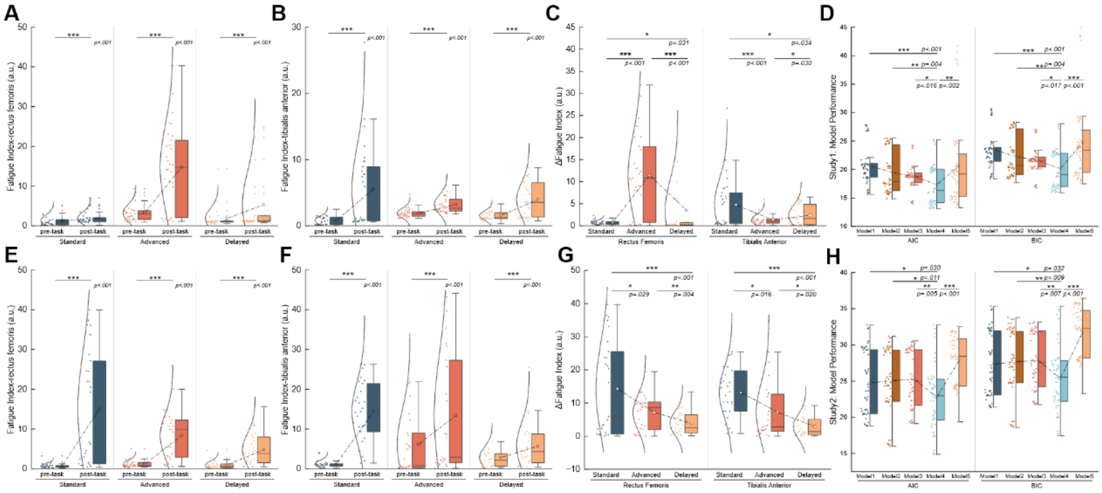

Figure S1. The results of the robustness validation.

All the results are consistent with our findings.

A, B, C, D: The verification results of Study 1.

E, F, G, H: The verification results of Study 2.

A: Changes in the fatigue index of rectus femoris in temporal modality. (Standard: p<.001, Cohen's d=1.37, 95%CI=[0.52, 0.87], Advanced: p<.001, Cohen's d=1.25, 95%CI=[8.27, 14.85], Delayed: p<.001, Cohen's d=0.50, 95%CI=[1.30, 6.10])

B: Changes in the fatigue index of tibialis anterior in temporal modality. (Standard: p<.001, Cohen's d=0.80, 95%CI=[2.85, 6.96], Advanced: p<.001, Cohen's d=1.07, 95%CI=[0.93, 1.80], Delayed: p<.001, Cohen's d=1.04, 95%CI=[1.59, 3.21])

C: Comparison of the changes of muscle fatigue level in temporal modality. (Rectus Femoris: Standard vs. Advanced: p<.001, Cohen's d=-1.74, 95%CI=[-14.76, -7.88], Standard vs. Delayed: p=.031, Cohen's d=-0.58, 95%CI=[-5.55, -0.58], Advanced vs. Delayed: p<.001, Cohen's d=1.02, 95%CI=[4.39, 12.42], Tibialis Anterior: Standard vs. Advanced: p<.001, Cohen's d=0.82, 95%CI=[1.56, 5.82], Standard vs. Delayed: p=.034, Cohen's d=0.54, 95%CI=[0.44, 4.84], Advanced vs. Delayed: p=.030, Cohen's d=-0.60, 95%CI=[-2.10, -0.21])

D: Comparison of model performance in temporal modality. (AIC: Model1 vs. Model4: p<.001, Cohen's d=0.98, 95%CI=[1.95, 4.87], Model2 vs. Model4: p=.004, Cohen's d=0.59, 95%CI=[0.79, 3.56], Model3 vs. Model4: p=.010, Cohen's d=0.49, 95%CI=[0.28, 2.70], Model5 vs. Model4: p=.002, Cohen's d=0.60, 95%CI=[1.31, 6.02], BIC: Model1 vs. Model4: p<.001, Cohen's d=0.98, 95%CI=[2.08, 4.83], Model2 vs. Model4: p=.004, Cohen's d=0.59, 95%CI=[0.68, 3.49], Model3 vs. Model4: p=.017, Cohen's d=0.49, 95%CI=[0.36, 2.73], Model5 vs. Model4: p<.001, Cohen's d=0.84, 95%CI=[2.80, 7.28])

E: Changes in the fatigue index of rectus femoris in spatial modality. (Standard: p<.001, Cohen's d=0.92, 95%CI=[8.22, 18.59], Advanced: p<.001, Cohen's d=1.36, 95%CI=[5.44, 9.51], Delayed: p<.001, Cohen's d=1.05, 95%CI=[2.47, 5.10])

F: Changes in the fatigue index of tibialis anterior in spatial modality. (Standard: p<.001, Cohen's d=1.79, 95%CI=[10.48, 16.03], Advanced: p<.001, Cohen's d=0.80, 95%CI=[4.16, 10.86], Delayed: p<.001, Cohen's d=0.79, 95%CI=[1.67, 4.38])

G: Comparison of the changes of muscle fatigue level in spatial modality. (Rectus Femoris: Standard vs. Advanced: p=.020, Cohen's d=0.57, 95%CI=[0.43, 11.99], Standard vs. Delayed: p<.001, Cohen's d=1.01, 95%CI=[5.25, 16.03], Advanced vs. Delayed: p=.004, Cohen's d=0.98, 95%CI=[2.01, 6.60], Tibialis Anterior: Standard vs. Advanced: p=.010, Cohen's d=0.70, 95%CI=[1.19, 10.05], Standard vs. Delayed: p<.001, Cohen's d=1.76, 95%CI=[7.27, 13.24], Advanced vs. Delayed: p=.020, Cohen's d=0.65, 95%CI=[00.67, 8.42])

H: Comparison of model performance in spatial modality. (AIC: Model1 vs. Model4: p=.030, Cohen's d=0.44, 95%CI=[0.25, 3.84], Model2 vs. Model4: p=.011, Cohen's d=0.54, 95%CI=[0.70, 4.17], Model3 vs. Model4: p=.005, Cohen's d=0.57, 95%CI=[0.87, 4.28], Model5 vs. Model4: p<.001, Cohen's d=1.26, 95%CI=[3.62, 6.78], BIC: Model1 vs. Model4: p=.032, Cohen's d=0.44, 95%CI=[0.27, 3.80], Model2 vs. Model4: p=.009, Cohen's d=0.54, 95%CI=[0.68, 4.27], Model3 vs. Model4: p=.007, Cohen's d=0.57, 95%CI=[0.64, 4.24], Model5 vs. Model4: p<.001, Cohen's d=1.58, 95%CI=[4.85, 7.94])

Data S1 The model performance of Model Group 5 in Study 1

Forms of model	$\overline{AIC}$	$\overline{BIC}$	SD
SF=aMF*TE+bTE+cHR	28.82	40.48	7.23
SF=aMF*TE+bTE+cHR^2	29.88	41.54	8.94
SF=aMF*TE+bTE+c/HR	27.63	39.29	7.02
SF=aMF*TE+bTE+c*exp(HR)	28.94	40.6	9.34
SF=aMF*TE+bTE^2+cHR	28.27	39.92	7.18
SF=aMF*TE+bTE^2+cHR^2	28.43	40.09	8.98
SF=aMF*TE+bTE^2+c/HR	28.02	39.67	7.15
SF=aMF*TE+bTE^2+c*exp(HR)	32.78	44.44	12.88
SF=aMF*TE+b*exp(TE)+cHR	27.85	39.51	8.02
SF=aMF*TE+b*exp(TE)+cHR^2	29.64	41.29	11.89
SF=aMF*TE+b*exp(TE)+c/HR	28.32	39.98	8.26
SF=aMF*TE+b*exp(TE)+c*exp(HR)	30.25	41.91	12.30
SF=aMF*TE^2+bTE+cHR	27.75	39.41	6.79
SF=aMF*TE^2+bTE+cHR^2	29.82	41.48	8.34
SF=aMF*TE^2+bTE+c/HR	28.76	40.42	8.14
SF=aMF*TE^2+bTE+c*exp(HR)	30.51	42.17	10.83
SF=aMF*TE^2+bTE^2+cHR	30.72	42.38	7.89
SF=aMF*TE^2+bTE^2+cHR^2	29.15	40.81	7.97
SF=aMF*TE^2+bTE^2+c/HR	28.07	39.73	7.72
SF=aMF*TE^2+bTE^2+c*exp(HR)	33.75	45.41	13.59
SF=aMF*TE^2+b*exp(TE)+cHR	28.81	40.47	8.08
SF=aMF*TE^2+b*exp(TE)+cHR^2	34.84	46.5	18.74
SF=aMF*TE^2+b*exp(TE)+c*exp(HR)	30.08	41.74	11.35
SF=a(MF*TE)+bTE+cHR	28.2	39.86	7.46
SF=a(MF*TE)+bTE+cHR^2	31.09	42.75	9.54
SF=a(MF*TE)+bTE+c/HR	27.63	39.28	7.65
SF=a(MF*TE)+bTE+c*exp(HR)	31.34	43	11.22
SF=a(MF*TE)+bTE^2+cHR	28.66	40.32	8.31
SF=a(MF*TE)+bTE^2+cHR^2	29.44	41.1	9.87
SF=a(MF*TE)+bTE^2+c/HR	28.12	39.77	6.93
SF=a(MF*TE)+bTE^2+c*exp(HR)	29.29	40.95	10.59
SF=a(MF*TE)+b*exp(TE)+cHR	28.07	39.73	6.36
SF=a(MF*TE)+b*exp(TE)+cHR^2	29.52	41.18	10.64
SF=a(MF*TE)+b*exp(TE)+c/HR	28.99	40.65	8.94
SF=a(MF*TE)+b*exp(TE)+c*exp(HR)	29.76	41.42	10.92
SF=aMF*exp(TE)+bTE+cHR	28.34	39.99	6.55
SF=aMF*exp(TE)+bTE+cHR^2	29.43	41.09	8.61

Forms of model	$\overline{AIC}$	$\overline{BIC}$	SD
SF=aMF*exp(TE)+bTE+c/HR	29.16	40.82	8.07
SF=aMF*exp(TE)+bTE+c*exp(HR)	32.44	44.1	12.57
SF=aMF*exp(TE)+bTE^2+cHR	29.39	41.05	8.17
SF=aMF*exp(TE)+bTE^2+cHR^2	28.56	40.22	8.09
SF=aMF*exp(TE)+bTE^2+c/HR	27.9	39.56	6.68
SF=aMF*exp(TE)+bTE^2+c*exp(HR)	28.43	40.09	10.89
SF=aMF*exp(TE)+b*exp(TE)+cHR	28.31	39.97	7.78
SF=aMF*exp(TE)+b*exp(TE)+cHR^2	32.39	44.04	17.36
SF=aMF*exp(TE)+b*exp(TE)+c*exp(HR)	30.81	42.47	12.74
SF=aMF*exp(TE)+b*exp(TE)+c/HR	28.32	39.98	7.51
SF=aMF^2*TE+bTE+cHR	27.37	39.03	5.43
SF=aMF^2*TE+bTE+cHR^2	28.73	40.39	8.22
SF=aMF^2*TE+bTE+c/HR	27.51	39.17	6.17
SF=aMF^2*TE+bTE+c*exp(HR)	28.66	40.32	11.23
SF=aMF^2*TE+bTE^2+cHR	28.57	40.23	8.03
SF=aMF^2*TE+bTE^2+cHR^2	35.48	47.14	18.32
SF=aMF^2*TE+bTE^2+c/HR	28.19	39.85	7.20
SF=aMF^2*TE+bTE^2+c*exp(HR)	29.3	40.96	10.46
SF=aMF^2*TE+b*exp(TE)+cHR	30.03	41.69	9.5
SF=aMF^2*TE+b*exp(TE)+cHR^2	31.76	43.42	13.71
SF=aMF^2*TE+b*exp(TE)+c/HR	27.96	39.62	7.80
SF=aMF^2*TE+b*exp(TE)+c*exp(HR)	30.6	42.26	10.67
SF=aMF^2*TE^2+bTE+cHR	27.67	39.33	5.92
SF=aMF^2*TE^2+bTE+cHR^2	28.31	39.97	9.72
SF=aMF^2*TE^2+bTE+c/HR	27.88	39.54	5.96
SF=aMF^2*TE^2+bTE+c*exp(HR)	28.22	39.88	9.38
SF=aMF^2*TE^2+bTE^2+cHR	29.09	40.75	7.56
SF=aMF^2*TE^2+bTE^2+cHR^2	31.2	42.86	14.47
SF=aMF^2*TE^2+bTE^2+c/HR	27.91	39.57	7.24
SF=aMF^2*TE^2+bTE^2+c*exp(HR)	32.72	44.38	13.55
SF=aMF^2*TE^2+b*exp(TE)+cHR	29.69	41.35	8.32
SF=aMF^2*TE^2+b*exp(TE)+cHR^2	30.74	42.39	9.78
SF=aMF^2*TE^2+b*exp(TE)+c/HR	28.79	40.45	9.45
SF=aMF^2*TE^2+b*exp(TE)+c*exp(HR)	30.62	42.28	11.34
SF=aMF^2/TE+bTE+cHR	27.84	39.5	7.18
SF=aMF^2/TE+bTE+cHR^2	28.57	40.23	9.16
SF=aMF^2/TE+bTE+c/HR	28.01	39.67	7.35

Forms of model	$\overline{AIC}$	$\overline{BIC}$	SD
SF=aMF^2/TE+bTE+c*exp(HR)	30.08	41.74	11.08
SF=aMF^2/TE+bTE^2+cHR	28.2	39.86	7.75
SF=aMF^2/TE+bTE^2+cHR^2	30.26	41.91	12.36
SF=aMF^2/TE+bTE^2+c/HR	28.43	40.09	6.81
SF=aMF^2/TE+bTE^2+c*exp(HR)	30.26	41.92	12.06
SF=aMF^2/TE+b*exp(TE)+cHR	28.4	40.06	9.04
SF=aMF^2/TE+b*exp(TE)+cHR^2	30.4	42.06	12.62
SF=aMF^2/TE+b*exp(TE)+c/HR	27.96	39.62	8.68
SF=aMF^2/TE+b*exp(TE)+c*exp(HR)	29.25	40.91	11
SF=aMF^2*exp(TE)+bTE+cHR	27.8	39.46	7.20
SF=aMF^2*exp(TE)+bTE+cHR^2	31.08	42.74	9.17
SF=aMF^2*exp(TE)+bTE+c/HR	28.48	40.14	7.36
SF=aMF^2*exp(TE)+bTE+c*exp(HR)	28.45	40.11	10.46
SF=aMF^2*exp(TE)+bTE^2+cHR	28.84	40.5	8.35
SF=aMF^2*exp(TE)+bTE^2+cHR^2	29.73	41.39	9.48
SF=aMF^2*exp(TE)+bTE^2+c/HR	28.11	39.77	6.74
SF=aMF^2*exp(TE)+bTE^2+c*exp(HR)	29.1	40.75	10.7
SF=aMF^2*exp(TE)+b*exp(TE)+cHR	29.78	41.43	9.09
SF=aMF^2*exp(TE)+b*exp(TE)+cHR^2	31.41	43.07	11.83
SF=aMF^2*exp(TE)+b*exp(TE)+c/HR	29.79	41.45	8.78
SF=aMF^2*exp(TE)+b*exp(TE)+c*exp(HR)	31.09	42.75	12.11
SF=a/MF*TE+bTE+cHR	30.34	42	12.18
SF=a/MF*TE+bTE+cHR^2	31.74	43.4	12.58
SF=a/MF*TE+bTE+c/HR	30.06	41.72	12.22
SF=a/MF*TE+bTE+c*exp(HR)	32.02	43.68	13.8
SF=a/MF*TE+bTE^2+cHR	30.68	42.33	12.59
SF=a/MF*TE+bTE^2+cHR^2	29.28	40.94	12.03
SF=a/MF*TE+bTE^2+c/HR	29.59	41.25	12.22
SF=a/MF*TE+bTE^2+c*exp(HR)	31.44	43.1	13.69
SF=a/MF*TE+b*exp(TE)+cHR	29.77	41.42	12.26
SF=a/MF*TE+b*exp(TE)+cHR^2	36.88	48.53	18.46
SF=a/MF*TE+b*exp(TE)+c/HR	29.51	41.17	12.77
SF=a/MF*TE+b*exp(TE)+c*exp(HR)	32.08	43.74	14.8
SF=a/MF*TE^2+bTE+cHR	27.7	39.36	6.54
SF=a/MF*TE^2+bTE+cHR^2	29.31	40.97	8.42
SF=a/MF*TE^2+bTE+c/HR	29.57	41.23	8.62
SF=a/MF*TE^2+bTE+c*exp(HR)	31.97	43.63	11.99

Forms of model	$\overline{AIC}$	$\overline{BIC}$	SD
SF=a/MF*TE^2+bTE^2+cHR	28.07	39.72	6.29
SF=a/MF*TE^2+bTE^2+cHR^2	29.74	41.4	10.76
SF=a/MF*TE^2+bTE^2+c/HR	27.54	39.2	6.00
SF=a/MF*TE^2+bTE^2+c*exp(HR)	30.95	42.61	11.45
SF=a/MF*TE^2+b*exp(TE)+cHR	28.37	40.03	6.38
SF=a/MF*TE^2+b*exp(TE)+cHR^2	30.89	42.54	12.6
SF=a/MF*TE^2+b*exp(TE)+c/HR	28.97	40.63	8.93
SF=a/MF*TE^2+b*exp(TE)+c*exp(HR)	31.76	43.42	11.98
SF=a/(MF*TE)+bTE+cHR	28.4	40.06	7.4
SF=a/(MF*TE)+bTE+cHR^2	28.26	39.92	8.9
SF=a/(MF*TE)+bTE+c/HR	29.46	41.11	9.24
SF=a/(MF*TE)+bTE+c*exp(HR)	30.09	41.75	11.47
SF=a/(MF*TE)+bTE^2+cHR	28.14	39.8	7.67
SF=a/(MF*TE)+bTE^2+cHR^2	31.59	43.25	12.09
SF=a/(MF*TE)+bTE^2+c/HR	27.82	39.48	7.35
SF=a/(MF*TE)+bTE^2+c*exp(HR)	28.48	40.14	10.07
SF=a/(MF*TE)+b*exp(TE)+cHR	28.72	40.38	8.65
SF=a/(MF*TE)+b*exp(TE)+cHR^2	31.39	43.04	13.99
SF=a/(MF*TE)+b*exp(TE)+c/HR	29.28	40.93	8.99
SF=a/(MF*TE)+b*exp(TE)+c*exp(HR)	29.96	41.62	10.49
SF=a/MF*exp(TE)+bTE+cHR	27.62	39.28	5.68
SF=a/MF*exp(TE)+bTE+cHR^2	30.09	41.75	8.5
SF=a/MF*exp(TE)+bTE+c/HR	28.58	40.24	6.9
SF=a/MF*exp(TE)+bTE+c*exp(HR)	29.45	41.11	10.08
SF=a/MF*exp(TE)+bTE^2+cHR	28.15	39.81	6.28
SF=a/MF*exp(TE)+bTE^2+cHR^2	28.54	40.2	8.15
SF=a/MF*exp(TE)+bTE^2+c/HR	27.31	38.96	5.18
SF=a/MF*exp(TE)+bTE^2+c*exp(HR)	28.35	40	9.46
SF=a/MF*exp(TE)+b*exp(TE)+cHR	30.04	41.7	8.18
SF=a/MF*exp(TE)+b*exp(TE)+cHR^2	33.18	44.84	17.64
SF=a/MF*exp(TE)+b*exp(TE)+c/HR	27.57	39.22	7.16
SF=a/MF*exp(TE)+b*exp(TE)+c*exp(HR)	30.07	41.73	10.53
SF=a*exp(MF)*TE+bTE+cHR^2	27.68	39.33	7.88
SF=a*exp(MF)*TE+bTE+c/HR	29.02	40.67	7.53
SF=a*exp(MF)*TE+bTE+cHR	27.61	39.27	5.75
SF=a*exp(MF)*TE+bTE+c*exp(HR)	31.24	42.9	12.6
SF=a*exp(MF)*TE+bTE^2+cHR	27.64	39.29	5.42

Forms of model	$\overline{AIC}$	$\overline{BIC}$	SD
SF=a*exp(MF)*TE+bTE^2+cHR^2	30.02	41.67	12.84
SF=a*exp(MF)*TE+bTE^2+c/HR	27.47	39.13	5.21
SF=a*exp(MF)*TE+bTE^2+c*exp(HR)	29.85	41.51	10.15
SF=a*exp(MF)*TE+b*exp(TE)+cHR	28.64	40.3	7.49
SF=a*exp(MF)*TE+b*exp(TE)+cHR^2	28.44	40.1	11.00
SF=a*exp(MF)*TE+b*exp(TE)+c/HR	28.49	40.15	6.94
SF=a*exp(MF)*TE+b*exp(TE)+c*exp(HR)	30.07	41.73	10.11
SF=a*exp(MF)*TE^2+bTE+cHR	25.68	37.34	4.37
SF=a*exp(MF)*TE^2+bTE+cHR^2	28.73	40.39	9.6
SF=a*exp(MF)*TE^2+bTE+c/HR	27.53	39.19	5.11
SF=a*exp(MF)*TE^2+bTE+c*exp(HR)	29.36	41.02	10.51
SF=a*exp(MF)*TE^2+bTE^2+cHR	27.22	38.88	4.96
SF=a*exp(MF)*TE^2+bTE^2+cHR^2	30.72	42.38	12.43
SF=a*exp(MF)*TE^2+bTE^2+c/HR	27.7	39.36	5.29
SF=a*exp(MF)*TE^2+bTE^2+c*exp(HR)	29.31	40.97	11.43
SF=a*exp(MF)*TE^2+b*exp(TE)+cHR	27.72	39.38	5.13
SF=a*exp(MF)*TE^2+b*exp(TE)+cHR^2	28.58	40.23	7.67
SF=a*exp(MF)*TE^2+b*exp(TE)+c/HR	27.55	39.21	5.39
SF=a*exp(MF)*TE^2+b*exp(TE)+c*exp(HR)	30.78	42.44	11.60
SF=a*exp(MF)/TE+bTE+cHR	28.08	39.73	7.53
SF=a*exp(MF)/TE+bTE+cHR^2	28.65	40.31	9.44
SF=a*exp(MF)/TE+bTE+c/HR	28.71	40.37	9.32
SF=a*exp(MF)/TE+bTE+c*exp(HR)	29.46	41.12	10.65
SF=a*exp(MF)/TE+bTE^2+cHR	28.89	40.55	8.68
SF=a*exp(MF)/TE+bTE^2+cHR^2	31.94	43.6	11.39
SF=a*exp(MF)/TE+bTE^2+c/HR	28.57	40.23	8.24
SF=a*exp(MF)/TE+bTE^2+c*exp(HR)	31.41	43.07	11.33
SF=a*exp(MF)/TE+b*exp(TE)+cHR	29.44	41.1	9.98
SF=a*exp(MF)/TE+b*exp(TE)+cHR^2	33.01	44.67	13.59
SF=a*exp(MF)/TE+b*exp(TE)+c/HR	30.82	42.48	10.45
SF=a*exp(MF)/TE+b*exp(TE)+c*exp(HR)	33.09	44.75	13.21
SF=a*exp(MF)*exp(TE)+bTE+cHR	28.14	39.8	8.31
SF=a*exp(MF)*exp(TE)+bTE+cHR^2	28.62	40.28	9.44
SF=a*exp(MF)*exp(TE)+bTE+c/HR	28.61	40.27	9.03
SF=a*exp(MF)*exp(TE)+bTE+c*exp(HR)	29.45	41.1	10.97
SF=a*exp(MF)*exp(TE)+bTE^2+cHR	27.7	39.36	6.62
SF=a*exp(MF)*exp(TE)+bTE^2+cHR^2	29.47	41.13	8.83

Forms of model	$\overline{AIC}$	$\overline{BIC}$	SD
SF=a*exp(MF)*exp(TE)+bTE^2+c/HR	27.98	39.64	7.46
SF=a*exp(MF)*exp(TE)+bTE^2+c*exp(HR)	29.62	41.27	10.67
SF=a*exp(MF)*exp(TE)+b*exp(TE)+cHR	29.51	41.17	9.76
SF=a*exp(MF)*exp(TE)+b*exp(TE)+cHR^2	29.72	41.38	11.06
SF=a*exp(MF)*exp(TE)+b*exp(TE)+c/HR	28.66	40.32	9.57
SF=a*exp(MF)*exp(TE)+b*exp(TE)+c*exp(HR)	30.43	42.09	12.08
SF=aMF*TE+b/TE+cHR	26.22	37.88	7.61
SF=aMF*TE+b/TE+cHR^2	28.59	40.25	10.44
SF=aMF*TE+b/TE+c/HR	29.68	41.34	6.53
SF=aMF*TE+b/TE+c*exp(HR)	27.68	39.34	7.81
SF=aMF*TE^2+b/TE+cHR	26.74	38.40	3.54
SF=aMF*TE^2+b/TE+cHR^2	28.51	40.17	10.04
SF=aMF*TE^2+b/TE+c/HR	26.55	38.21	11.06
SF=aMF*TE^2+b/TE+c*exp(HR)	27.55	39.21	13.55
SF=aMF*/TE+b/TE+cHR	27.71	39.37	9.96
SF=aMF*/TE+b/TE+cHR^2	30.21	41.87	5.77
SF=aMF*/TE+b/TE+c/HR	28.31	39.97	5.57
SF=aMF*/TE+b/TE+c*exp(HR)	28.87	40.53	6.34
SF=aMF*exp(TE)+b/TE+cHR	30.7	42.36	3.76
SF=aMF*exp(TE)+b/TE+cHR^2	29.31	40.97	4.04
SF=aMF*exp(TE)+b/TE+c/HR	28.92	40.58	5.52
SF=aMF*exp(TE)+b/TE+c*exp(HR)	27.85	39.51	5.40
SF=aMF^2*TE+b/TE+cHR	29.45	41.11	11.03
SF=aMF^2*TE+b/TE+cHR^2	27.76	39.42	4.07
SF=aMF^2*TE+b/TE+c/HR	28.17	39.83	13.12
SF=aMF^2*TE+b/TE+c*exp(HR)	26.19	37.85	7.98
SF=aMF^2*TE^2+b/TE+cHR	27.04	38.70	8.88
SF=aMF^2*TE^2+b/TE+cHR^2	27.29	38.95	11.03
SF=aMF^2*TE^2+b/TE+c/HR	28.24	39.90	5.15
SF=aMF^2*TE^2+b/TE+c*exp(HR)	28.26	39.92	11.46
SF=aMF^2*/TE+b/TE+cHR	26.69	38.35	9.48
SF=aMF^2*/TE+b/TE+cHR^2	29.55	41.21	11.88
SF=aMF^2*/TE+b/TE+c/HR	26.02	37.68	8.77
SF=aMF^2*/TE+b/TE+c*exp(HR)	29.82	41.48	3.22
SF=aMF^2*exp(TE)+b/TE+cHR	26.19	37.85	2.91
SF=aMF^2*exp(TE)+b/TE+cHR^2	28.11	39.77	13.63
SF=aMF^2*exp(TE)+b/TE+c/HR	28.22	39.88	10.52

Forms of model	$\overline{AIC}$	$\overline{BIC}$	SD
SF=aMF^2*exp(TE)+b/TE+c*exp(HR)	30.68	42.34	8.63
SF=a/MF*TE+b/TE+cHR	28.89	40.55	6.39
SF=a/MF*TE+b/TE+cHR^2	27.29	38.95	8.85
SF=a/MF*TE+b/TE+c/HR	27.96	39.62	9.81
SF=a/MF*TE+b/TE+c*exp(HR)	27.94	39.60	10.52
SF=a/MF*TE^2+b/TE+cHR	26.23	37.89	7.13
SF=a/MF*TE^2+b/TE+cHR^2	28.2	39.86	10.01
SF=a/MF*TE^2+b/TE+c/HR	30.77	42.43	4.89
SF=a/MF*TE^2+b/TE+c*exp(HR)	28.24	39.90	8.54
SF=a/MF*/TE+b/TE+cHR	27.72	39.38	3.76
SF=a/MF*/TE+b/TE+cHR^2	27.98	39.64	10.13
SF=a/MF*/TE+b/TE+c/HR	30.4	42.06	6.53
SF=a/MF*/TE+b/TE+c*exp(HR)	30.07	41.73	7.58
SF=a/MF*exp(TE)+b/TE+cHR	28.68	40.34	11.65
SF=a/MF*exp(TE)+b/TE+cHR^2	28.78	40.44	9.14
SF=a/MF*exp(TE)+b/TE+c/HR	28	39.66	12.84
SF=a/MF*exp(TE)+b/TE+c*exp(HR)	27.84	39.50	8.88
SF=a*exp(MF)*TE+b/TE+cHR^2	28.18	39.84	11.94
SF=a*exp(MF)*TE+b/TE+c/HR	28.04	39.70	3.68
SF=a*exp(MF)*TE+b/TE+cHR	28.81	40.47	10.8
SF=a*exp(MF)*TE+b/TE+c*exp(HR)	28.41	40.07	3.00
SF=a*exp(MF)*TE^2+b/TE+cHR	30.66	42.32	5.15
SF=a*exp(MF)*TE^2+b/TE+cHR^2	28.91	40.57	2.86
SF=a*exp(MF)*TE^2+b/TE+c/HR	29.33	40.99	7.38
SF=a*exp(MF)*TE^2+b/TE+c*exp(HR)	30.1	41.76	6.87
SF=a*exp(MF)*/TE+b/TE+cHR	26.62	38.28	5.57
SF=a*exp(MF)*/TE+b/TE+cHR^2	29.53	41.19	7.75
SF=a*exp(MF)*/TE+b/TE+c/HR	26.41	38.07	10.47
SF=a*exp(MF)*/TE+b/TE+c*exp(HR)	27.15	38.81	6.65
SF=a*exp(MF)*exp(TE)+b/TE+cHR	26.27	37.93	5.09
SF=a*exp(MF)*exp(TE)+b/TE+cHR^2	26.91	38.57	6.68
SF=a*exp(MF)*exp(TE)+b/TE+c/HR	30.92	42.58	12.64
SF=a*exp(MF)*exp(TE)+b/TE+c*exp(HR)	29.44	41.10	9.05

Data S2 The model performance of Model Group 5 in Study 2

Forms of model	$\overline{AIC}$	$\overline{BIC}$	SD
SF=aMF*SE+bSE+cHR	21.82	29.59	3.10
SF=aMF*SE+bSE+cHR^2	21.80	29.57	2.88
SF=aMF*SE+bSE+c/HR	21.73	29.51	3.34
SF=aMF*SE+bSE+c*exp(HR)	21.34	29.11	3.52
SF=aMF*SE+bSE^2+cHR	21.62	29.39	3.79
SF=aMF*SE+bSE^2+cHR^2	21.49	29.27	2.99
SF=aMF*SE+bSE^2+c/HR	21.73	29.51	4.44
SF=aMF*SE+bSE^2+c*exp(HR)	21.56	29.33	3.32
SF=aMF*SE+b*exp(SE)+cHR	21.36	29.13	3.22
SF=aMF*SE+b*exp(SE)+cHR^2	21.84	29.61	3.68
SF=aMF*SE+b*exp(SE)+c/HR	22.01	29.78	4.82
SF=aMF*SE+b*exp(SE)+c*exp(HR)	21.37	29.14	3.52
SF=aMF*SE^2+bSE+cHR	22.07	29.84	2.78
SF=aMF*SE^2+bSE+cHR^2	21.33	29.10	2.99
SF=aMF*SE^2+bSE+c/HR	21.54	29.31	3.06
SF=aMF*SE^2+bSE+c*exp(HR)	21.58	29.35	3.19
SF=aMF*SE^2+bSE^2+cHR	21.93	29.70	3.25
SF=aMF*SE^2+bSE^2+cHR^2	21.82	29.59	4.76
SF=aMF*SE^2+bSE^2+c/HR	22.07	29.84	4.52
SF=aMF*SE^2+bSE^2+c*exp(HR)	21.66	29.43	4.19
SF=aMF*SE^2+b*exp(SE)+cHR	21.83	29.61	4.51
SF=aMF*SE^2+b*exp(SE)+cHR^2	21.91	29.68	3.76
SF=aMF*SE^2+b*exp(SE)+c*exp(HR)	22.05	29.82	3.12
SF=aMF/SE+bSE+cHR	21.41	29.19	3.97
SF=aMF/SE+bSE+cHR^2	22.56	30.33	3.60
SF=aMF/SE+bSE+c/HR	21.96	29.73	4.58
SF=aMF/SE+bSE+c*exp(HR)	21.60	29.38	3.62
SF=aMF/SE+bSE^2+cHR	21.85	29.62	4.30
SF=aMF/SE+bSE^2+cHR^2	21.46	29.23	3.14
SF=aMF/SE+bSE^2+c/HR	22.24	30.01	4.75
SF=aMF/SE+bSE^2+c*exp(HR)	22.01	29.78	3.62
SF=aMF/SE+b*exp(SE)+cHR	21.99	29.76	3.96
SF=aMF/SE+b*exp(SE)+cHR^2	21.45	29.22	3.61
SF=aMF/SE+b*exp(SE)+c/HR	22.47	30.24	5.90
SF=aMF/SE+b*exp(SE)+c*exp(HR)	22.04	29.81	4.08
SF=aMF*exp(SE)+bSE+cHR	21.33	29.10	2.94
SF=aMF*exp(SE)+bSE+cHR^2	21.57	29.34	2.54

Forms of model	$\overline{\text{AIC}}$	$\overline{\text{BIC}}$	SD
SF=aMF*exp(SE)+bSE+c/HR	20.05	31.39	4.56
SF=aMF*exp(SE)+bSE+c*exp(HR)	21.36	29.13	2.88
SF=aMF*exp(SE)+bSE^2+cHR	21.35	29.12	3.27
SF=aMF*exp(SE)+bSE^2+cHR^2	21.83	29.60	2.95
SF=aMF*exp(SE)+bSE^2+c/HR	21.31	29.08	2.86
SF=aMF*exp(SE)+bSE^2+c*exp(HR)	21.35	29.12	2.98
SF=aMF*exp(SE)+b*exp(SE)+cHR	21.43	29.21	2.46
SF=aMF*exp(SE)+b*exp(SE)+cHR^2	21.54	29.31	3.49
SF=aMF*exp(SE)+b*exp(SE)+c*exp(HR)	21.30	29.07	2.99
SF=aMF*exp(SE)+b*exp(SE)+c/HR	21.48	29.25	3.69
SF=aMF^2*SE+bSE+cHR	21.62	29.40	3.82
SF=aMF^2*SE+bSE+cHR^2	21.39	29.17	3.59
SF=aMF^2*SE+bSE+c/HR	21.73	29.50	4.04
SF=aMF^2*SE+bSE+c*exp(HR)	21.56	29.33	3.36
SF=aMF^2*SE+bSE^2+cHR	22.07	29.84	3.83
SF=aMF^2*SE+bSE^2+cHR^2	21.92	29.70	4.45
SF=aMF^2*SE+bSE^2+c/HR	21.51	29.29	3.76
SF=aMF^2*SE+bSE^2+c*exp(HR)	22.08	29.85	4.41
SF=aMF^2*SE+b*exp(SE)+cHR	21.77	29.55	3.61
SF=aMF^2*SE+b*exp(SE)+cHR^2	22.03	29.80	4.91
SF=aMF^2*SE+b*exp(SE)+c/HR	21.65	29.43	3.93
SF=aMF^2*SE+b*exp(SE)+c*exp(HR)	22.11	29.89	3.87
SF=aMF^2*SE^2+bSE+cHR	21.60	29.37	4.47
SF=aMF^2*SE^2+bSE+cHR^2	22.44	30.21	4.45
SF=aMF^2*SE^2+bSE+c/HR	21.88	29.65	4.36
SF=aMF^2*SE^2+bSE+c*exp(HR)	22.02	29.80	4.69
SF=aMF^2*SE^2+bSE^2+cHR	22.33	30.10	4.60
SF=aMF^2*SE^2+bSE^2+cHR^2	21.87	29.64	5.18
SF=aMF^2*SE^2+bSE^2+c/HR	21.27	29.04	3.69
SF=aMF^2*SE^2+bSE^2+c*exp(HR)	21.86	29.64	4.91
SF=aMF^2*SE^2+b*exp(SE)+cHR	21.68	29.45	4.76
SF=aMF^2*SE^2+b*exp(SE)+cHR^2	22.14	29.92	5.08
SF=aMF^2*SE^2+b*exp(SE)+c/HR	22.10	29.87	5.21
SF=aMF^2*SE^2+b*exp(SE)+c*exp(HR)	22.08	29.85	4.99
SF=aMF^2/SE+bSE+cHR	21.54	29.31	3.74
SF=aMF^2/SE+bSE+cHR^2	22.00	29.77	4.13
SF=aMF^2/SE+bSE+c/HR	21.69	29.47	4.07

Forms of model	$\overline{AIC}$	$\overline{BIC}$	SD
SF=aMF^2/SE+bSE+c*exp(HR)	22.11	29.89	4.18
SF=aMF^2/SE+bSE^2+cHR	21.79	29.56	4.53
SF=aMF^2/SE+bSE^2+cHR^2	21.45	29.22	3.92
SF=aMF^2/SE+bSE^2+c/HR	21.72	29.50	4.88
SF=aMF^2/SE+bSE^2+c*exp(HR)	22.25	30.03	4.72
SF=aMF^2/SE+b*exp(SE)+cHR	21.58	29.35	4.58
SF=aMF^2/SE+b*exp(SE)+cHR^2	22.00	29.78	4.88
SF=aMF^2/SE+b*exp(SE)+c/HR	22.03	29.80	5.53
SF=aMF^2/SE+b*exp(SE)+c*exp(HR)	22.40	30.17	5.68
SF=aMF^2*exp(SE)+bSE+cHR	22.17	29.94	3.89
SF=aMF^2*exp(SE)+bSE+cHR^2	22.28	30.06	4.44
SF=aMF^2*exp(SE)+bSE+c/HR	22.26	30.04	4.16
SF=aMF^2*exp(SE)+bSE+c*exp(HR)	21.47	29.24	5.02
SF=aMF^2*exp(SE)+bSE^2+cHR	21.44	29.21	4.02
SF=aMF^2*exp(SE)+bSE^2+cHR^2	21.78	29.56	3.48
SF=aMF^2*exp(SE)+bSE^2+c/HR	21.57	29.34	3.90
SF=aMF^2*exp(SE)+bSE^2+c*exp(HR)	21.50	29.27	3.15
SF=aMF^2*exp(SE)+b*exp(SE)+cHR	21.52	29.30	4.11
SF=aMF^2*exp(SE)+b*exp(SE)+cHR^2	21.73	29.50	5.04
SF=aMF^2*exp(SE)+b*exp(SE)+c/HR	22.08	29.85	4.15
SF=aMF^2*exp(SE)+b*exp(SE)+c*exp(HR)	21.98	29.76	3.91
SF=a/MF*SE+bSE+cHR	24.12	31.89	9.98
SF=a/MF*SE+bSE+cHR^2	23.24	31.01	8.63
SF=a/MF*SE+bSE+c/HR	23.98	31.75	10.05
SF=a/MF*SE+bSE+c*exp(HR)	23.04	30.82	10.33
SF=a/MF*SE+bSE^2+cHR	22.85	30.62	7.84
SF=a/MF*SE+bSE^2+cHR^2	22.00	29.77	6.02
SF=a/MF*SE+bSE^2+c/HR	23.16	30.94	7.18
SF=a/MF*SE+bSE^2+c*exp(HR)	22.10	29.87	6.16
SF=a/MF*SE+b*exp(SE)+cHR	22.42	30.20	7.68
SF=a/MF*SE+b*exp(SE)+cHR^2	22.21	29.98	6.92
SF=a/MF*SE+b*exp(SE)+c/HR	22.38	30.16	7.93
SF=a/MF*SE+b*exp(SE)+c*exp(HR)	25.31	33.08	9.98
SF=a/MF*SE^2+bSE+cHR	23.40	31.17	10.40
SF=a/MF*SE^2+bSE+cHR^2	22.93	30.70	10.22
SF=a/MF*SE^2+bSE+c/HR	23.43	31.20	10.77
SF=a/MF*SE^2+bSE+c*exp(HR)	25.28	33.05	12.30

Forms of model	$\overline{AIC}$	$\overline{BIC}$	SD
SF=a/MF*SE^2+bSE^2+cHR	23.98	31.75	9.45
SF=a/MF*SE^2+bSE^2+cHR^2	22.68	30.46	8.80
SF=a/MF*SE^2+bSE^2+c/HR	21.94	29.71	6.28
SF=a/MF*SE^2+bSE^2+c*exp(HR)	22.83	30.61	9.86
SF=a/MF*SE^2+b*exp(SE)+cHR	24.34	32.12	10.76
SF=a/MF*SE^2+b*exp(SE)+cHR^2	23.31	31.09	7.93
SF=a/MF*SE^2+b*exp(SE)+c/HR	23.14	30.91	9.26
SF=a/MF*SE^2+b*exp(SE)+c*exp(HR)	23.36	31.13	8.62
SF=a/(MF*SE)+bSE+cHR	22.22	29.99	8.03
SF=a/(MF*SE)+bSE+cHR^2	21.89	29.66	5.84
SF=a/(MF*SE)+bSE+c/HR	22.81	30.58	8.61
SF=a/(MF*SE)+bSE+c*exp(HR)	22.57	30.34	9.06
SF=a/(MF*SE)+bSE^2+cHR	23.27	31.04	7.30
SF=a/(MF*SE)+bSE^2+cHR^2	22.37	30.14	8.07
SF=a/(MF*SE)+bSE^2+c/HR	22.83	30.60	8.97
SF=a/(MF*SE)+bSE^2+c*exp(HR)	23.40	31.17	8.97
SF=a/(MF*SE)+b*exp(SE)+cHR	22.93	30.71	8.30
SF=a/(MF*SE)+b*exp(SE)+cHR^2	22.57	30.35	7.28
SF=a/(MF*SE)+b*exp(SE)+c/HR	22.35	30.13	8.94
SF=a/(MF*SE)+b*exp(SE)+c*exp(HR)	22.81	30.58	8.46
SF=a/MF*exp(SE)+bSE+cHR	24.70	32.47	11.98
SF=a/MF*exp(SE)+bSE+cHR^2	23.94	31.72	12.16
SF=a/MF*exp(SE)+bSE+c/HR	23.50	31.28	11.09
SF=a/MF*exp(SE)+bSE+c*exp(HR)	25.11	32.88	14.43
SF=a/MF*exp(SE)+bSE^2+cHR	26.22	33.99	13.25
SF=a/MF*exp(SE)+bSE^2+cHR^2	23.79	31.56	12.08
SF=a/MF*exp(SE)+bSE^2+c/HR	22.77	30.54	10.64
SF=a/MF*exp(SE)+bSE^2+c*exp(HR)	22.90	30.67	8.56
SF=a/MF*exp(SE)+b*exp(SE)+cHR	22.92	30.69	9.36
SF=a/MF*exp(SE)+b*exp(SE)+cHR^2	24.70	32.48	12.47
SF=a/MF*exp(SE)+b*exp(SE)+c/HR	24.57	32.34	11.92
SF=a/MF*exp(SE)+b*exp(SE)+c*exp(HR)	23.43	31.20	8.50
SF=a*exp(MF)*SE+bSE+cHR^2	21.64	29.41	3.49
SF=a*exp(MF)*SE+bSE+c/HR	22.33	30.10	3.63
SF=a*exp(MF)*SE+bSE+cHR	21.39	29.16	2.35
SF=a*exp(MF)*SE+bSE+c*exp(HR)	21.56	29.33	3.59
SF=a*exp(MF)*SE+bSE^2+cHR	21.41	29.18	3.04

Forms of model	$\overline{AIC}$	$\overline{BIC}$	SD
SF=a*exp(MF)*SE+bSE^2+cHR^2	21.36	29.13	3.39
SF=a*exp(MF)*SE+bSE^2+c/HR	22.11	29.88	4.80
SF=a*exp(MF)*SE+bSE^2+c*exp(HR)	21.62	29.39	3.20
SF=a*exp(MF)*SE+b*exp(SE)+cHR	21.96	29.73	3.28
SF=a*exp(MF)*SE+b*exp(SE)+cHR^2	21.58	29.35	4.03
SF=a*exp(MF)*SE+b*exp(SE)+c/HR	21.90	29.67	5.74
SF=a*exp(MF)*SE+b*exp(SE)+c*exp(HR)	21.58	29.35	3.39
SF=a*exp(MF)*SE^2+bSE+cHR	22.12	29.89	4.94
SF=a*exp(MF)*SE^2+bSE+cHR^2	22.13	29.90	4.32
SF=a*exp(MF)*SE^2+bSE+c/HR	21.87	29.64	4.93
SF=a*exp(MF)*SE^2+bSE+c*exp(HR)	21.99	29.77	4.42
SF=a*exp(MF)*SE^2+bSE^2+cHR	21.74	29.52	4.21
SF=a*exp(MF)*SE^2+bSE^2+cHR^2	21.97	29.74	4.66
SF=a*exp(MF)*SE^2+bSE^2+c/HR	21.81	29.58	4.75
SF=a*exp(MF)*SE^2+bSE^2+c*exp(HR)	21.57	29.34	3.80
SF=a*exp(MF)*SE^2+b*exp(SE)+cHR	21.65	29.42	4.80
SF=a*exp(MF)*SE^2+b*exp(SE)+cHR^2	22.54	30.31	3.99
SF=a*exp(MF)*SE^2+b*exp(SE)+c/HR	21.57	29.34	5.27
SF=a*exp(MF)*SE^2+b*exp(SE)+c*exp(HR)	22.33	30.10	3.67
SF=a*exp(MF)/SE+bSE+cHR	21.39	29.17	3.48
SF=a*exp(MF)/SE+bSE+cHR^2	21.50	29.28	3.99
SF=a*exp(MF)/SE+bSE+c/HR	22.04	29.81	4.32
SF=a*exp(MF)/SE+bSE+c*exp(HR)	21.41	29.18	3.04
SF=a*exp(MF)/SE+bSE^2+cHR	22.09	29.86	3.99
SF=a*exp(MF)/SE+bSE^2+cHR^2	21.72	29.49	3.95
SF=a*exp(MF)/SE+bSE^2+c/HR	21.77	29.54	4.94
SF=a*exp(MF)/SE+bSE^2+c*exp(HR)	21.49	29.26	3.77
SF=a*exp(MF)/SE+b*exp(SE)+cHR	21.98	29.76	4.10
SF=a*exp(MF)/SE+b*exp(SE)+cHR^2	21.94	29.71	4.72
SF=a*exp(MF)/SE+b*exp(SE)+c/HR	22.00	29.78	4.64
SF=a*exp(MF)/SE+b*exp(SE)+c*exp(HR)	21.99	29.76	3.94
SF=a*exp(MF)*exp(SE)+bSE+cHR	21.81	29.58	3.92
SF=a*exp(MF)*exp(SE)+bSE+cHR^2	21.94	29.71	4.79
SF=a*exp(MF)*exp(SE)+bSE+c/HR	21.77	29.55	4.99
SF=a*exp(MF)*exp(SE)+bSE+c*exp(HR)	21.70	29.47	4.72
SF=a*exp(MF)*exp(SE)+bSE^2+cHR	21.61	29.39	4.86
SF=a*exp(MF)*exp(SE)+bSE^2+cHR^2	21.68	29.46	3.73

Forms of model	$\overline{AIC}$	$\overline{BIC}$	SD
SF=a*exp(MF)*exp(SE)+bSE^2+c/HR	21.50	29.27	3.38
SF=a*exp(MF)*exp(SE)+bSE^2+c*exp(HR)	21.44	29.21	3.27
SF=a*exp(MF)*exp(SE)+b*exp(SE)+cHR	21.68	29.45	5.19
SF=a*exp(MF)*exp(SE)+b*exp(SE)+cHR^2	21.92	29.69	4.66
SF=a*exp(MF)*exp(SE)+b*exp(SE)+c/HR	21.81	29.58	4.81
SF=a*exp(MF)*exp(SE)+b*exp(SE)+c*exp(HR)	21.93	29.70	4.01
